# Structural characterization, derivatization, and bioactivities of secondary metabolites produced by termite-associated *Streptomyces lannensis* BYF-106

**DOI:** 10.1128/spectrum.01818-24

**Published:** 2025-04-15

**Authors:** Jun Wu, Miao Zhang, Jian Tao, MengRu Liu, JianHao Xiong, TaoShan Jiang, YaXuan Wang, XiaoHong Li, YueYue Li, CaiPing Yin, ShuXiang Zhang, XinHua Liu, YingLao Zhang

**Affiliations:** 1 School of Life Science, Anhui Agricultural University, Hefei, China; 2 Center for Biological Science and Technology, Advanced Institute of Natural Sciences, Beijing Normal University, Zhuhai, China; 3 School of Pharmacy, Anhui Medical University, Hefei, China; University of Mississippi, University, Mississippi, USA

**Keywords:** *Odontotermes formosanus*, *Streptomyces lannensis*, natural products, antibacterial activity, anti-inflammatory activity, cytotoxic activity

## Abstract

Two new C-glycoside angucycline-related analogs, urdamycin Y (**1**) and grincamycin W (**2**), as well as eight known metabolites (**3–10**), were identified from termite-associated *Streptomyces lannensis* BYF-106 based on global natural products social molecular networking (GNPS). The putative biosynthetic pathways of urdamycin Y (**1**) and grincamycin W (**2**) were proposed using bioinformatic analysis of the full genome of *S. lannensis* BYF-106. In addition, four new derivative compounds (**4A**, **5A**, **6A,** and **6B**) were synthesized via acetylation and methylation, respectively. Partial compounds were evaluated *in vitro* for antibacterial, anti-inflammatory, and cytotoxic activities. Vineomycinone B2 (**3**), fridamycin D (**4**), and 6-hydroxytetrangulol (**5**) displayed broad-spectrum antibacterial activities against *S. aureus*, methicillin-resistant *S. aureus,* and *P. syringae* pv. *actinidae*. Furthermore, urdamycin Y (**1**) exhibited potent inhibition on NO production, with an IC_50_ value of 4.8 *µ*M, which was comparable to that of Bay11-7082 with an IC_50_ value of 2.1 µM. Subsequently, the possible anti-inflammatory mechanism of urdamycin Y (**1**) was explored by molecular docking simulation. Finally, most of the tested metabolites showed significant cytotoxic activities against HCT-116, HT-29, and A375. Notably, 6-hydroxytetrangulol (**5**) and the acetyl derivative **5A** showed extremely strong cytotoxic activities against HCT-116, with IC_50_ values of 9.8 and 2.2 µM, respectively. Moreover, **5A** showed extremely strong cytotoxic activity against A375 (IC_50_ <0.2 µM), and the conceivable cytotoxic activity mechanism was also proposed by molecular docking. These findings indicated metabolites of insect-associated *S. lannensis* BYF-106 might be a potential source for developing new bioactive drugs in food, agriculture, and biomedical fields.

**IMPORTANCE:**

Frequent attention to soil microorganisms has led to the rediscovery of known compounds. By contrast, insect-associated *Streptomyces* have been shown to produce a more diverse array of unique bioactive secondary metabolites compared to soil *Streptomyces*. In our ongoing effort to explore structurally diverse bioactive natural products from termite-associated *Streptomyces*, we discovered that the strain *S. lannensis* BYF-106 exhibited potent bioactivity. Chemical investigation of BYF-106 resulted in the isolation of two new C-glycoside angucycline-related analogs: urdamycin Y (**1**) and grincamycin W (**2**). In addition, four new derivative compounds (**4A**, **5A**, **6A**, and **6B**) were synthesized through acetylation and methylation, respectively. Urdamycin Y (**1**) exhibited a strong inhibitory effect on NO production, and most of the tested metabolites showed significant cytotoxic activity. These findings indicate that the metabolites of BYF-106 may offer promising avenues for the exploration and development of new bioactive drugs.

## INTRODUCTION

Microbial natural products play an irreplaceable role in food, agriculture, and biomedical fields for their complex chemical structures and unique biological activities (1, 2). Natamycin, a natural antifungal agent belonging to the class of polyene macrolides, is produced by the soil-derived *Streptomyces natalensis* (3). Frenolicin B, a pyranonaphthoquinone identified from the soil-derived *Streptomyces* sp. NEAU-H3, has the potential to be used as a fungicide agent for controlling the disease of wheat caused by *Fusarium graminearum* (4). Pteridic acid H, a novel spiroketal polyketide identified from the soil-derived *Streptomyces iranensis* HM 35, could effectively promote root growth in *Arabidopsis* under abiotic stress (5). However, the frequent attention to soil microorganisms led to the rediscovery of known compounds (6). To accelerate the discovery of new bioactive drugs, there is a pressing need to explore novel microbial resources.

Insect-associated *Streptomyces* have been proven to produce unique bioactive secondary metabolites compared to soil *Streptomyces* (7). For instance, two novel pentacyclic polyketides, fasamycin D and E, produced from the plant-ants-associated *Streptomyces formicae* and exhibited potent antibacterial activities against multiple pathogenic bacteria (*Bacillus subtilis*, methicillin-resistant *Staphylococcus aureus*, and vancomycin-resistant *Enterococcus faecium*) (8). Phoslactomycin K, a novel polyketide *δ*-lactone obtained from an Australian wasp nest-derived *Streptomyces* sp. CMB-MW079, displayed strong antifungal activities against *Fusarium pseudograminearum* 64,952a and *Colletotrichum acutatum* (9). Cyphomycin, caniferolide C, and GT-35, three macrolides isolated from fungus-growing ants associated with *Streptomyces* sp. ISID311, showed potent antiprotozoal activity against *Leishmania donovani* (10).

Recently, our research has focused on the investigation of bioactive natural products from *Streptomyces* associated with fungus-farming termite *Odontotermes formosanus* (11–17). During our ongoing effort to discover new bioactive metabolites from fungus-farming termite-derived *Streptomyces*, we discovered the strain *Streptomyces lannensis* BYF-106 exhibited potent antibacterial and antifungal activities. Chemical investigation into BYF-106 resulted in the isolation of two C-glycoside angucycline derivatives, named fridamycin D and grincamycin N (18). Members of the angucycline group are type II polyketide-derived metabolites that exhibit structural diversity, primarily characterized by variations in the oxidation degrees of the core skeleton and the position and type of substitutions (19). This extensive structural diversity gives rise to a broad range of biological activities, including prominent antitumor and antibacterial properties (20). Therefore, the secondary metabolites of BYF-106 warrant further investigation, with the potential to uncover novel active metabolites that could serve as scaffolds for novel therapeutic drug development.

Here, two new C-glycoside angucycline-related metabolites, named urdamycin Y (**1**) and grincamycin W (**2**), along with other known metabolites (**3–10**) were further isolated from BYF-106 based on global natural products social molecular networking (GNPS). Meanwhile, the plausible biosynthetic pathways of urdamycin Y (**1**) and grincamycin W (**2**) were proposed. Subsequently, four new derivative compounds were synthesized by chemical modification. Finally, the antibacterial, anti-inflammatory, and cytotoxic activities of these compounds were also investigated.

## MATERIALS AND METHODS

### General experimental procedures

Silica gel (200–300 mesh) for column chromatography and Sephadex LH-20 were purchased from Qingdao Marine Chemical Factory, China. UV spectra were acquired using a UV-2550 spectrophotometer. IR spectra were obtained on a Nicolet spectrophotometer (Thermo Scientific, USA). HR-ESI-MS data were obtained using a G6230AA time-of-flight mass spectrometer (Agilent Technologies, USA). ^1^H, ^13^C-NMR, and 2D NMR spectra were recorded on an Agilent DD2 600 MHZ spectrometer (Agilent, USA) and the tetramethylsilane (TMS) was used as an internal standard. HPLC was used on a ^18^C column (SHIMAD, 4.6 × 150 mm).

### Actinomycete material and bioinformatic analysis

*S. lannensis* BYF-106 (GenBank No. AB562508) was isolated from body surfaces of *O. formosanus* collected from the suburb of Jiangyin City, Jiangsu province. The strain was deposited at the School of Life Sciences, Anhui Agricultural University, Hefei, China.

Whole-genome sequencing of BYF-106 was performed using a PacBio RS platform in combination with Illumina sequencing technology. The genome of BYF-106 was sequenced using PacBio RS combined with Illumina sequencing platforms. The biosynthetic gene clusters (BGCs) of secondary metabolites were identified using antiSMASH v6.0.1 and 2ndfind.

### Cultivation and extraction

The strain *S. lannensis* BYF-106 was cultivated in 100 mL of Gause’s No. 1 medium at 180 rpm. After 5 days of incubation at 28°C, 10 mL of cultured liquid was transferred into 400 mL Gause’s No. 1 medium in 1 L flasks (78 × 400 mL, total volume 31.2 L) and incubated at 180 rpm at 28°C for 7 days. A total of 31.2 L of fermentation broth was filtered to remove the bacterial cells, and the supernatant was extracted with EtOAc (3 × 15.6 L) at room temperature to obtain cultured extract (8.0 g).

### LC-MS/MS and molecular networking analysis

LC-MS/MS data were processed using the GNPS platform (http://gnps.ucsd.edu) for molecular networking analysis. All data are converted to .mzXML format files by MSConvert software. Molecular networking was performed using the GNPS data analysis workflow and the spectral clustering algorithm. The spectral networks were imported into Cytoscape (ver. 3.10.1) for visualization. The parameters of GNPS analysis were as follows: precursor ion mass tolerance, 0.02 Da; fragment ion mass tolerance, 0.02 Da; min pairs cosine, 0.6; minimum matched fragment ions, 2; minimum cluster size, 1.

### Purification of compounds 1–10

The crude extract was loaded to a silica-gel column using CH_2_Cl_2_-CH_3_OH (100:0–0:100 vol/vol) gradient elution to afford nine fractions (Fr1–Fr9). Fr1 was purified by recrystallization in methanol to yield **5** (2.3 mg) and two subfractions (Fr1-1 and Fr1-2). Subfraction Fr1-1 was subjected to a silica-gel column and further loaded onto a Sephadex LH-20 column (MeOH) to give **4** (6.5 mg) and **6** (1.2 mg). Fr1-2 was purified by HPLC (90% MeOH, 10% H_2_O with 0.1% acetic acid) to afford **2** (1.2 mg, *t*_R_ = 18 min). Fr3 was applied to a silica-gel column and a Sephadex LH-20 column (MeOH) to give **1** (4.9 mg) and **8** (3.5 mg). Fr4 was purified by a silica-gel column and a Sephadex LH-20 column (MeOH) to obtain three subfractions Fr4-1, Fr4-2, and Fr4-3. Subfraction Fr4-1 was purified by HPLC (90% MeOH, 10% H_2_O with 0.1% acetic acid) to obtain **3** (1.2 mg, *t*_R_ = 34 min). Subfraction Fr4-2 was purified by HPLC (80% MeOH, 10% H_2_O with 0.1% acetic acid) to obtain **9** (1.2 mg, *t*_R_ = 23 min) and **10** (1.2 mg, *t*_R_ = 27 min). Subfraction Fr4-3 was applied to a silica-gel column and a Sephadex LH-20 column (MeOH) to give **7** (2.1 mg).

Urdamycin Y (**1**): blue solid; UV(MeOH): *λ*_max_ nm (log *ε*): 560 (375.5), 234 (3419.32), 259 (279.92); IR *ν*_max_ (KBr, cm^−1^) 3440, 3135, 2963, 1637, 1400. ^1^H and ^13^C NMR data, see Table 1. HR-ESI-MS (negative) *m*/*z* 750.2195 [M-H]^-^ (calcd for C_41_H_36_NO_13_^-^, 750.2187).

**TABLE 1:** ^1^H (600 MHz) and ^13^C (150 MHz) NMR for urdamycin Y (**1**) in acetone-*d_6_*

Position	*δ*_C_	Type	*δ*_H_, mult (*J* in Hz)
1	207.0	C	
2	53.0	CH_2_	2.83 3.28, d (13.2)
3	76.0	C	
4	44.9	CH_2_	2.10 2.12
4a	81.3	C	
5	138.0	CH	6.10, d (10.0)
6	119.0	CH	6.99, d (9.9)
6a	124.3	C	
7	156.8	C	
7a	112.8	C	
8	187.5	C	
9	143.1	C	
10	136.5	CH	8.20, s
11	130.9	C	
11a	117.3	C	
12	141.6	C	
12a	129.4	C	
12b	79.2	C	
13	30.1	CH_3_	1.20, s
1′	72.0	CH	4.88, m
2′	37.9	CH_2_	1.42, m 2.37, m
3′	77.2	CH	3.85, m
4′	75.1	CH	3.31, d (8.8)
5′	74.9	CH	3.51, m
6′	17.6	CH_3_	1.03, d (6.0)
1′′	92.2	CH	5.19, d (2.4)
2′′	72.3	CH	4.39, d (2.4)
3′′	40.6	CH	2.50, m 2.81, m
4′′	208.5	C	
5′′	78.2	CH	4.65, dd (13.5, 6.8)
6′′	16.4	CH_3_	1.21, s
1′′′	159.6	C	
2′′′	128.1	C	
3′′′	109.2	C	
4′′′	133.0	CH	8.03, s
5′′′	137.6	C	
6′′′	113.1	CH	7.59, d (8.2)
7′′′	123.6	CH	7.26, m
8′′′	121.8	CH	7.15, m
9′′′	121.3	CH	7.59, d (8.2)
10′′′	128.1	C	
7-OH			13.27, brs
4′′′-NH			11.27, brs

### Derivatization of compounds 4, 5, and 6

According to the previous method (21), compounds **4**, **5,** and **6** were reacted with derivatization reagents to form three new acetylated derivatives (**4A**, **5A,** and **6A**). Briefly, compounds **4**, **5,** and **6** (0.08 mmol) were dissolved in 2 mL of dichloromethane, respectively. Subsequently, the sample solution was treated with acetic anhydride (Ac_2_O; 3.00 mmol) and 4-dimethylaminopyridine (DMAP; 0.5 mmol) for 24 h at 30°C. Compound **6** was reacted with derivatization reagents to form a new methylated derivative (**6B**). The method of methylation reaction (22) was as follows: **6** (0.08 mmol) mixed with 2 mL of dichloromethane was treated with 16 µL of potassium iodide (CH_3_I) and sodium hydride (NaH; 0.42 mmol) for 24 h at 30°C.

The progress of the reaction was continuously monitored through thin-layer chromatography (TLC) analysis until the reaction was complete. Finally, the reaction solution was successively extracted by EtOAc, concentrated by rotary distillation, and recrystallized in methanol. Compound **4A** (2.1 mg, *t*_R_ = 32 min) was gathered by high-performance liquid chromatography (HPLC; 90% MeOH, 10% H_2_O with 0.1% acetic acid). Compounds **5A** (1.4 mg), **6A** (2.5 mg), and **6B** (1.2 mg) were purified by a Sephadex LH-20 column (MeOH).

Compound **4A**: ^1^H NMR (600 MHz, DMSO-*d*_6_) *δ* 13.30 (1H, brs, 9-OH), 12.94 (1H, brs, 4-OH), 7.92 (1H, d, *J* = 6.8 Hz, H-2), 7.80 (1H, d, *J* = 6.2 Hz, H-1), 7.74 (1H, d, *J* = 7.2 Hz, H-6), 7.70 (1H, d, *J* = 5.4 Hz, H-7), 5.23 (1H, s, H-1″), 5.00 (1H, d, *J* = 10.7 Hz, H-1′), 4.71 (1H, d, *J* = 6.0 Hz, H-5″), 4.34 (1H, s, H-2″), 3.86 (1H, m, H-3′), 3.72 (1H, m, H-5′), 3.60 (3H, s, H-15), 3.51 (1H, m, H-4′), 3.28 (2H, d, *J* = 6.0 Hz, H-11), 3.11 (1H, d, *J* = 14.9 Hz, H-13a), 2.88 (2H, m, H-13b, H-3a″), 2.50 (1H, m, H-2a′), 2.46 (1H, s, H-3b″), 1.95 (3H, s, H-18), 1.63 (1H, dd, *J* = 22.4, 10.9 Hz, H-2b′), 1.42 (3H, s, H-16), 1.25 (3H, d, *J* = 6.0 Hz, H-6′), and 1.24 (3H, s, H-6″); ^13^C NMR (150 MHz, CDCl_3_) *δ* 208.5 (C, C-4″), 187.7 (C, C-5, C-10), 169.9 (C, C-17), 169.7 (C, C-14), 158.0 (C, C-4), 139.4 (CH, C-7), 137.0 (C, C-3), 133.6 (CH, C-2), 133.1 (C, C-8), 131.8 (C, C-10a), 131.6 (C, C-5a), 118.9 (CH, C-1), 118.4 (CH, C-6), 115.3 (C, C-4a, C-9a), 90.4 (CH, C-1″), 80.8 (C, C-12), 76.9 (CH, C-5″), 75.7 (CH, C-3′), 73.6 (CH, C-5′), 73.5 (CH, C-4′), 70.8 (CH, C-2″), 70.5 (CH, C-1′), 51.3 (OCH_3_, C-15), 41.8 (CH_2_, C-13), 40.1 (CH_2_, C-3″), 36.6 (CH_2_, C-11), 35.8 (CH_2_, C-2′), 23.7 (CH_3_, C-16), 22.0 (CH_3_, C-18), 17.3 (CH_3_, C-6′), and 16.0 (CH_3_, C-6″); HR-ESI-MS (negative) *m*/*z* 651.2070 [M-H]^-^ (calcd for C_34_H_35_O_13_^-^, 651.2078).

Compound **5A**: ^1^H NMR (600 MHz, CDCl_3_) *δ* 7.91 (1H, d, *J* = 7.5 Hz), 7.75 (1H, t, *J* = 8.5 Hz), 7.65 (1H, s), 7.49 (1H, s), 7.37 (1H, d, *J* = 7.9 Hz), 7.31 (1H, s), 2.55 (3H, s), 2.46 (3H, d, *J* = 1.5 Hz), 2.44 (3H, d, *J* = 1.4 Hz), 2.36 (3H, d, *J* = 1.4 Hz); ^13^C NMR (150 MHz, CDCl_3_) *δ* 185.2, 180.6, 169.9, 169.3, 168.5, 149.6, 147.2, 145.8, 141.2, 138.0, 137.0, 136.5, 134.8, 129.1, 127.1, 127.0, 125.8, 125.1, 125.0, 124.0, 120.0, 21.8, and 21.2; HR-ESI-MS (positive) *m*/*z* 469.0891 [M + Na]^+^ (calcd for C_25_H_18_O_8_Na^+^, 469.0899).

Compound **6A**: ^1^H NMR (600 MHz, CDCl_3_) *δ* 12.65 (1H, brs), 8.26 (1H, d, *J* = 8.3 Hz), 8.06 (1H, d, *J* = 8.5 Hz), 7.89 (1H, d, *J* = 7.6 Hz), 7.59 (2H, m), 7.34 (1H, s), 5.19 (1H, m), 5.01 (1H, d, *J* = 10.2 Hz), 4.75 (1H, d, *J* = 5.7 Hz), 4.35 (1H, m), 3.84 (1H, m), 3.60 (1H, m), 3.52 (1H, m), 2.66 (2H, m), 2.57 (3H, s), 2.47 (1H, m), 2.33 (3H, s), 1.56 (1H, m), 1.42 (3H, m), and 1.39 (3H, m); ^13^C NMR (150 MHz, CDCl_3_) *δ* 207.7, 188.7, 184.9, 168.9, 158.1, 147.5, 140.6, 138.6, 136.0, 135.1, 133.9, 133.8, 133.7, 126.2, 125.9, 122.0, 121.7, 118.5, 115.0, 91.6, 77.9, 77.1, 74.8, 71.6, 71.4, 40.1, 36.9, 21.7, 21.1, 17.7, and 16.3; HR-ESI-MS (positive) *m*/*z* 587.1908 [M + H]^+^ (calcd for C_33_H_31_O_10_^+^, 587.1917).

Compound **6B**: ^1^H NMR (600 MHz, CDCl_3_) *δ* 8.20 (1H, d, *J* = 8.4 Hz), 7.97 (1H, d, *J* = 7.6 Hz), 7.89 (2H, m), 7.26 (1H, m), 6.90 (1H, s), 5.19 (1H, s), 4.99 (1H, d, *J* = 9.7 Hz), 4.76 (1H, s), 4.34 (1H, m), 3.97 (6H, s), 3.82 (1H, m), 3.59 (1H, m), 3.52 (1H, m), 2.66 (2H, m), 2.54 (3H, s), 2.30 (1H, m), 1.62 (1H, m), 1.26 (3H, m), and 1.24 (3H, m); ^13^C NMR (150 MHz, CDCl_3_) *δ* 207.6, 185.7, 182.3, 157.6, 157.4, 156.8, 140.8, 140.7, 138.4, 138.2, 138.1, 133.9, 132.7, 132.6, 122.6, 122.5, 120.2, 111.5, 91.6, 77.2, 77.1, 74.8, 72.1, 71.4, 62.8, 56.2, 40.1, 37.8, 22.3, 17.7, and 16.3; HR-ESI-MS (positive) *m*/*z* 573.2115 [M + H]^+^ (calcd for C_33_H_33_O_9_^+^, 573.2124).

### Antibacterial bioassay

The disc diffusion method was applied to evaluate the antibacterial activity of compounds against *Staphylococcus aureus*, methicillin-resistant *S. aureus* (MRSA), *Pseudomonas syringae* pv. *actinidae*, *Xanthomonas oryzae* pv. *oryzae,* and *Xanthomonas oryzae* pv. *oryzicola* (23). The two tested bacterial strains (*S. aureus* and MRSA) were cultivated on LB medium at 37°C for 12 h. Subsequently, 0.1% bacterial cells were added to LB solid media plates. Finally, these filter paper disks with 5 µL of tested compounds (10 mg/mL) were added to LB solid media plates and incubated at 37°C for 24 h. The processes of tested compounds against *P. syringae* pv. *actinidae*, *Xanthomonas oryzae* pv. *oryzae,* and *Xanthomonas oryzae* pv. *oryzicola* were the same as the above steps, except for the incubation temperature (28°C). All experiments were performed in triplicates.

### NO inhibition bioassay

According to the previous method (24), RAW 264.7 cells were preincubated with test compounds for 1 h and subsequently treated with 1 µg/mL lipopolysaccharide (LPS) for 24 h. The Griess assay was applied to measure the nitric oxide (NO) production at 540 nm using a microplate reader. (E)−3-(4-Methylphenylsulfonyl)−2-propenenitrile (BAY 11–7082) was used as a positive control. All experiments were performed in triplicates.

### Cytotoxicity bioassay

Referring to the previous method (25), the MTT assay was used to assess the cytotoxic activities of tested compounds against the human colon cancer cell lines (HCT-116 and HT-29) and the human malignant melanoma cell lines (A375). SEL120-34A was used as a positive control, and the 50% cell growth inhibition (IC_50_) is shown in Table 5. Three parallel replicates were employed in all experiments.

### Molecular docking studies

The molecular docking studies were accomplished using the Discovery Studio 2019 software. Briefly, the structures of cyclooxygenase-2 (COX-2, PDB ID: 3LN1) and serine/threonine protein kinase (BRAF^V600E^, PDB ID: 2FB8) were obtained from the Protein Data Bank and preprocessed by Discovery Studio 2019 software. Then, compounds were drawn by ChemDraw 20.0 and preprocessed by Discovery Studio 2019 software. Finally, molecular docking was achieved by referring to the previously described method (26, 27).

## RESULTS

### Chemical composition analysis

**Fig 1:**
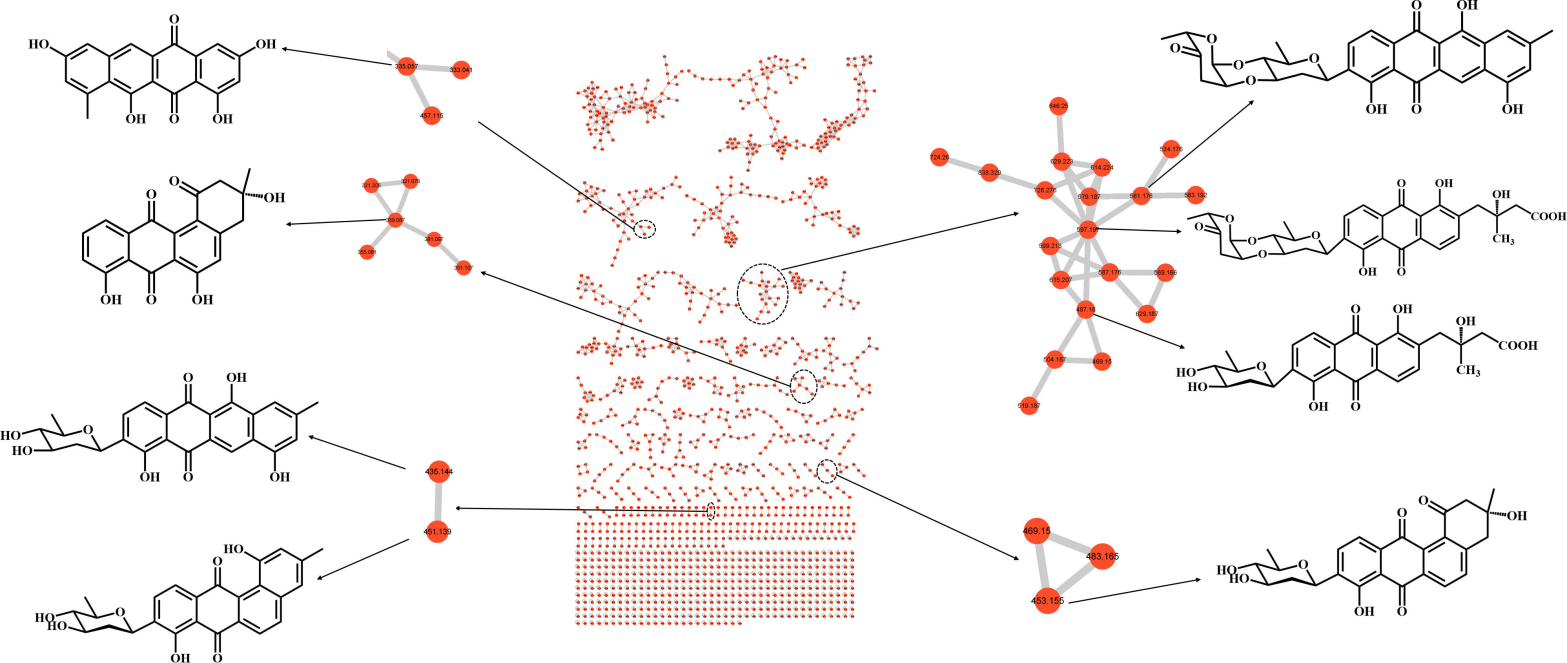
Molecular networking of metabolites from *S. lannensis* BYF-106 in gauze No. 1 medium.

The EtOAc extract derived from *S. lannensis* BYF-106 cultivated in Gause’s No. 1 medium was analyzed using high-resolution LC-MS/MS analysis. Raw LC-MS/MS data were processed using the GNPS platform. As shown in Fig. 1, the molecular networking detected a significant number of C-glycoside angucycline-related analogs, such as fridamycin D, vineomycinone B2, and grincamycin N. The C-glycoside angucyclines exhibited diverse bioactivities including anticancer and antibacterial activities (19). Therefore, the metabolites of BYF-106 in gauze No. 1 warrant further investigation.

### Structure elucidation

**Fig 2:**
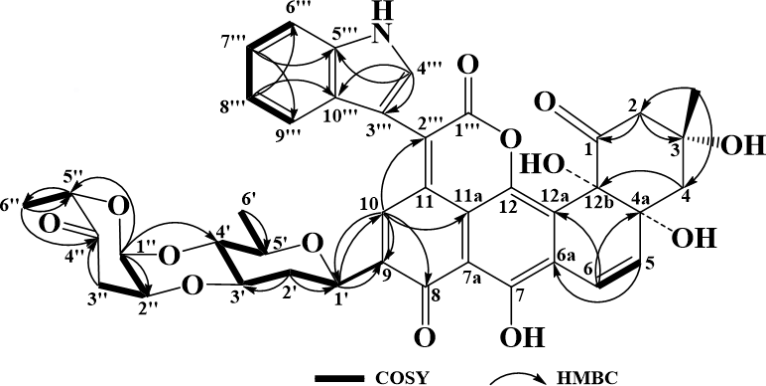
Key ^1^H−^1^H correlations by spectroscopy (^1^H-^1^H COSY) (lines) and HMBCs (arrows) of urdamycin Y (1).

Urdamycin Y (**1**) was obtained as a blue solid. Its molecular formula was determined as C_41_H_37_NO_13_ by HR-ESI-MS at *m*/*z* 750.2195 [M-H]^-^. The ^1^H NMR spectrum of **1** exhibited two olefinic hydrogen atom signals *δ*_H_ [6.10 (1H, d, *J* = 10.0 Hz) and 6.99 (1H, d, *J* = 9.9 Hz)], four aromatic hydrogen atom signals *δ*_H_ [7.15 (1H, m), 7.26 (1H, m), 7.59 (2H, d, *J* = 8.2 Hz), and 8.20 (1H, s)], three methyl group signals *δ*_H_ [1.03 (3H, d, *J* = 6.0 Hz), 1.20 (3H, s), and 1.21 (3H, s)] (Table 1). The ^13^C NMR spectrum showed 41 carbonyl carbons signals including two olefinic carbons signals (*δ*_C_ 119.0 and 138.0), five aromatic carbons signals (*δ*_C_ 136.5, 113.1, 123.6, 121.8, and 121.3), and three methyl carbons signals (*δ*_C_ 16.4, 17.6, and 30.1). The ^1^H and ^13^C NMR data suggested compound **1** shared the identical planar structure as urdamycin D, which possesses an angular tetracyclic ring system and the unique structural unit indole-pyranone (28). Furthermore, the ^1^H and ^13^C NMR spectroscopic data also indicated the presence of an *α*-cinerulose B-(1→4, 2→3)-*β*-olivosyl unit in compound **1**, which was the same as the previous reports (29). HMBC spectroscopy revealed correlations from the H-10 (*δ*_H_ 8.20) to C-8 (*δ*_C_ 187.5) and C-1′ (*δ*_C_ 72.0), from the H-1′ (*δ*_H_ 4.88) to C-9 (*δ*_C_ 143.1) and C-10 (*δ*_C_ 136.5), indicating a presence of the disaccharide at C-8 (Fig. 2; Fig. S2 to S9). Thus, the structure of **1** was solved and named urdamycin Y.

Grincamycin W (**2**) was isolated as a yellow solid. The molecular formula of **2** was established as C_31_H_30_O_11_ based on HR-ESI-MS (*m*/*z* 577.1724 [M-H]^-^). The ^1^H NMR spectrum showed three –OH signals *δ_H_* [4.08 (1H, brs), 10.16 (1H, brs), and 12.72 (1H, brs)], three aromatic hydrogen atom signals *δ*_H_ [7.58 (1H, d, *J* = 7.8 Hz), 7.78 (1H, s), and 7.92 (1H, d, *J* = 7.8 Hz)], two sets of methylene hydrogen signals *δ*_H_ [2.85 (1H, m), 3.07 (1H, d, *J* = 13.6 Hz), 3.00 (1H, d, *J* = 17.8 Hz), and 3.26 (1H, dd, *J* = 17.7, 1.1 Hz)], three methyl groups signals *δ*_H_ [1.49 (3H, s), 1.27 (3H, d, *J* = 6.7 Hz), and 1.33 (3H, d, *J* = 6.0 Hz)] (Table 2). The ^13^C NMR spectrum showed eleven aromatic carbon signals (*δ*_C_ 129.1, 160.4, 113.3, 136.9, 116.3, 159.1, 135.8, 134.8, 119.5, 135.8, and 137.8), three carbonyl group signals (*δ*_C_ 182.3, 189.6, and 208.6), and three methyl carbon signals (*δ*_C_ 16.7, 18.1, and 30.9). The ^1^H and ^13^C NMR spectroscopic data also revealed that the *α*-cinerulose B-(1→4, 2→3)-*β*-olivosyl unit was in the presence of compound **2**. These data were identical to that of grincamycin M, a natural product reported from the culture broth of *Streptomyces* sp. XZHG99T (30). The most significant difference between compound **2** and grincamycin M was the absence of the methoxy group at C-5 and the presence of an additional hydroxyl group (Fig. S10 to S12). In addition, the HR-ESI-MS of compound **2** was in good agreement with that of the calculated value (C_31_H_29_O_11_^-^ 577.1710, err 2.4 ppm). Consequently, the structure of **2** was named grincamycin W.

**TABLE 2:** ^1^H (600 MHz) and ^13^C (150 MHz) NMR for grincamycin W (**2**) in acetone-*d_6_*

Position	*δ*_C_	Type	*δ*_H_, mult (*J* in Hz)
1	197.1	C	
2	54.2	CH_2_	2.85, m 3.07, d (13.6)
3	72.7	C	
4	38.8	CH_2_	3.00, d (17.8) 3.26, dd (17.7, 1.1)
4a	129.1	C	
5	160.8	C	
6	108.2	CH	7.78, s
6a	136.9	C	
7	189.6	C	
7a	116.3	C	
8	159.1	C	
9	135.8	C	
10	134.8	CH	7.92, d (7.8)
11	119.5	CH	7.58, d (7.8)
11a	134.8	C	
12	182.3	C	
12a	135.8	C	
12b	137.8	C	
13	30.9	CH_3_	1.49, s
1′	72.6	CH	5.03, m
2′	37.8	CH_2_	1.61, m 2.38, m
3′	77.7	CH	3.89, m
4′	75.5	CH	3.58, m
5′	75.4	CH	3.62, m
6′	18.1	CH_3_	1.33, d (6.0)
1′′	92.5	CH	5.25, d (2.7)
2′′	72.3	CH	4.43, m
3′′	40.9	CH	2.51, d (3.4) 2.54, d (3.6)
4′′	208.6	C	
5′′	78.5	CH	4.77, dd (13.4, 6.7)
6′′	16.7	CH_3_	1.27, d (6.7)
3-OH			4.08, brs
5-OH			10.16, brs
8-OH			12.72, brs

**Fig 3:**
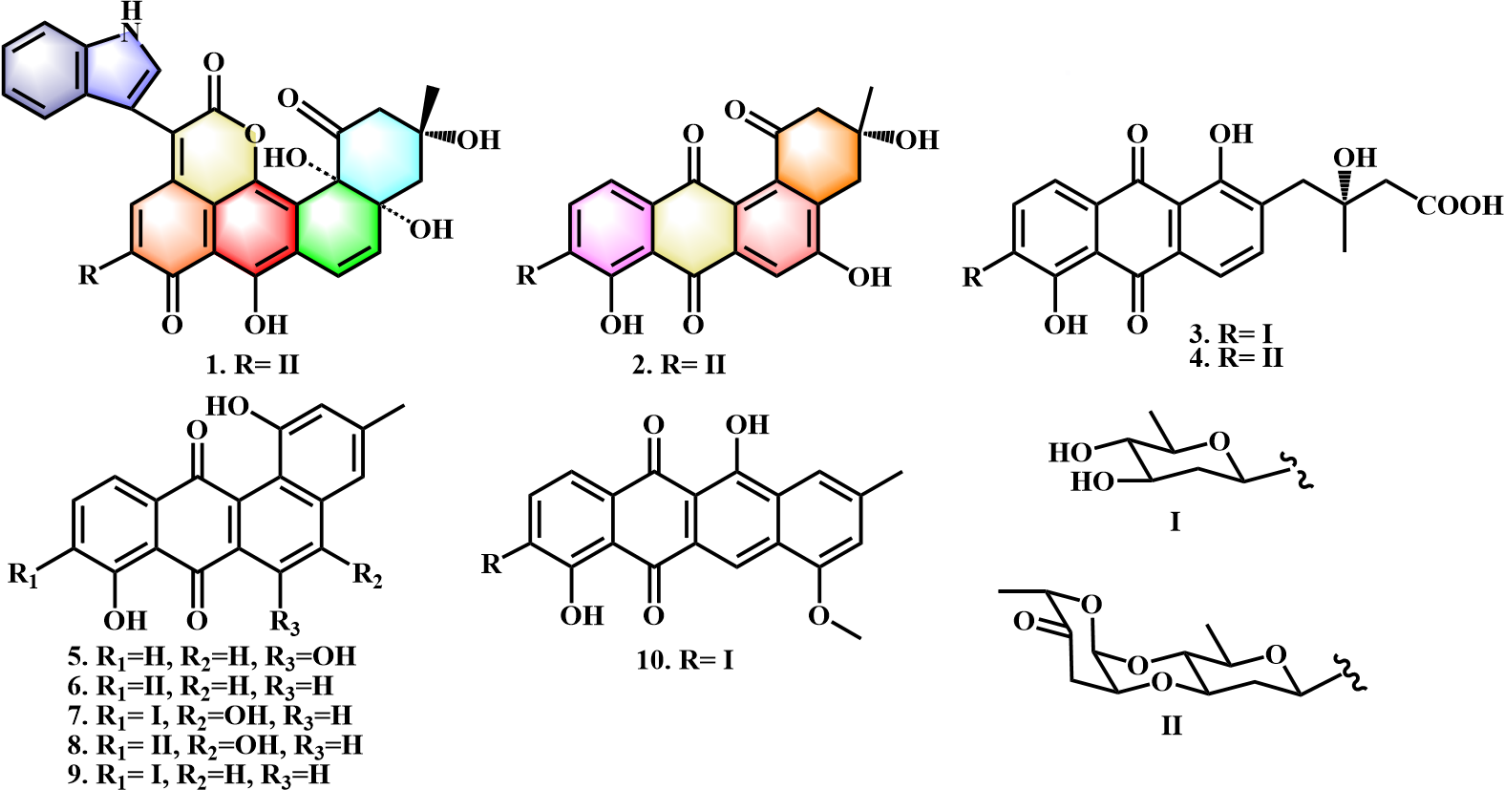
Chemical structures of secondary metabolites 1–10 of *S. lannensis* BYF-106.

The other secondary metabolites were identified as vineomycinone B2 (**3**) (31), fridamycin D (**4**) (18), 6-hydroxytetrangulol (**5**) (32), marangucycline B (**6**) (33), 4-hydroxyl-dehydroxyaquayamycin (**7**) (34), landomycin N (**8**) (35), dehydroxyaquayamycin (**9**) (33), and 7-O-methylgaltamycinone (**10**) (31) by spectroscopic analysis and comparison of their NMR data in the literature (Fig. 3).

### Proposed biosynthetic pathway of compounds 1 and 2

The classic structural characteristics of **1** and **2** defined these compounds as a polyketide derived from a modular type II polyketide synthase (PKS) biosynthetic pathway. To identify the biosynthetic gene clusters (BGCs) of compounds **1** and **2** in BYF-106, the genome of the strain was analyzed using antiSMASH v7.7.0. Genome mining revealed a candidate biosynthetic gene cluster (*UDM*) with 97% similarity to the galtamycin C BGC. This 113.531 kb *UDM* BGC contained 108 open reading frames (ORFs) including the PKS core enzymes responsible for the generation of the initial angucycline or angucyclinone backbone (UDM75, UDM76, and UDM77), four oxidases (UDM50, UDM51, UDM82, and UDM84), three glycosyltransferases (UDM67, UDM69, and UDM70) (36, 37) (Table S1).

**Fig 4:**
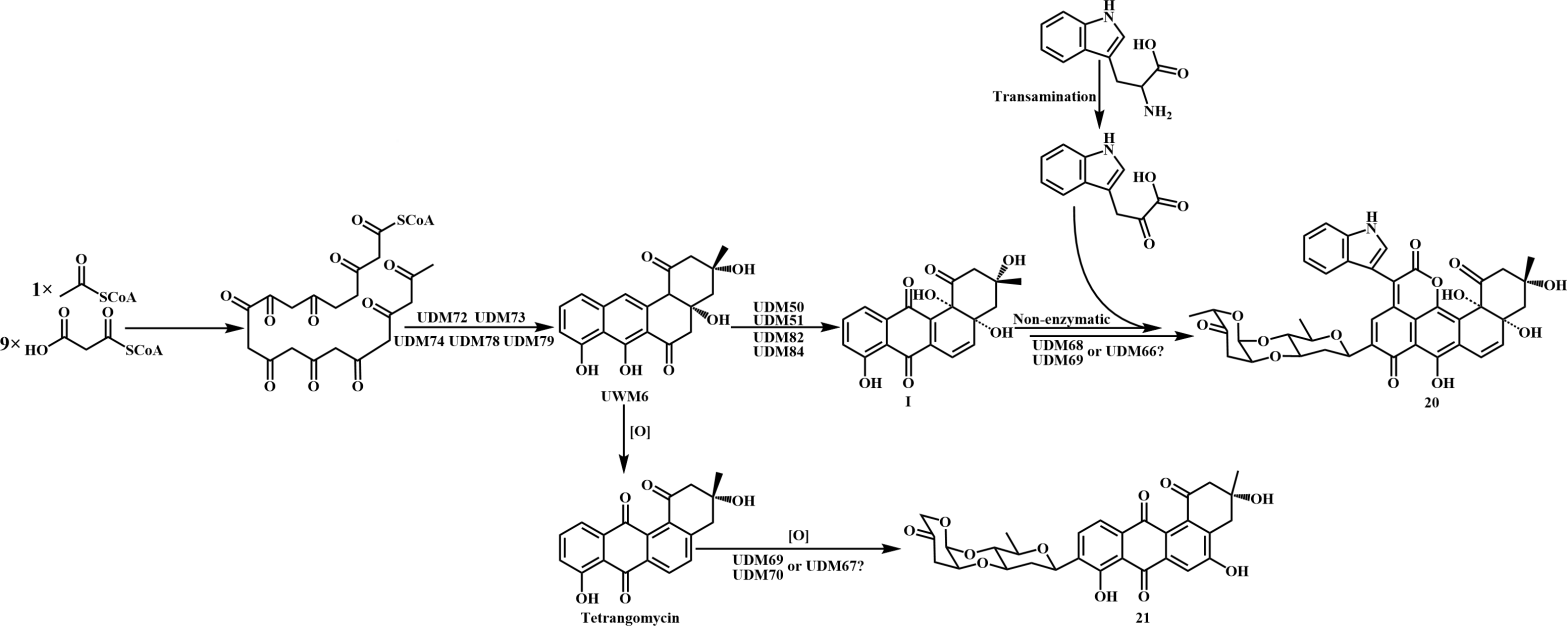
Proposed biosynthetic pathway for secondary metabolites 1 and 2 from *S. lannensis* BYF-106.

As shown in Fig. 4, the putative biosynthetic pathway for compounds **1** and **2** was proposed. First, one acetyl CoA molecule and nine malonyl CoA molecules were continuously catalyzed to form an intermediate product (UWM6) by UDM75, UDM76, UDM77, UDM50, UDM51, UDM82, and UDM84. UWM6 was continuously oxidized by UDM50, UDM51, UDM82, and UDM84 to form an intermediate I (36). Then, the intermediate I was glycosylated by glycosyltransferases (UDM67/UDM69 or UDM70). In addition, indole-3-pyruvate formed a lactone ring at the C-11 and C-12 positions through a non-enzymatic reaction to form compound **1** (38). In another branch, UWM6 was continuously oxidized and subsequently glycosylated by UDM67/UDM69 or UDM70 to form compound **2**.

### Derivatization of compounds 4–6

**Fig 5:**
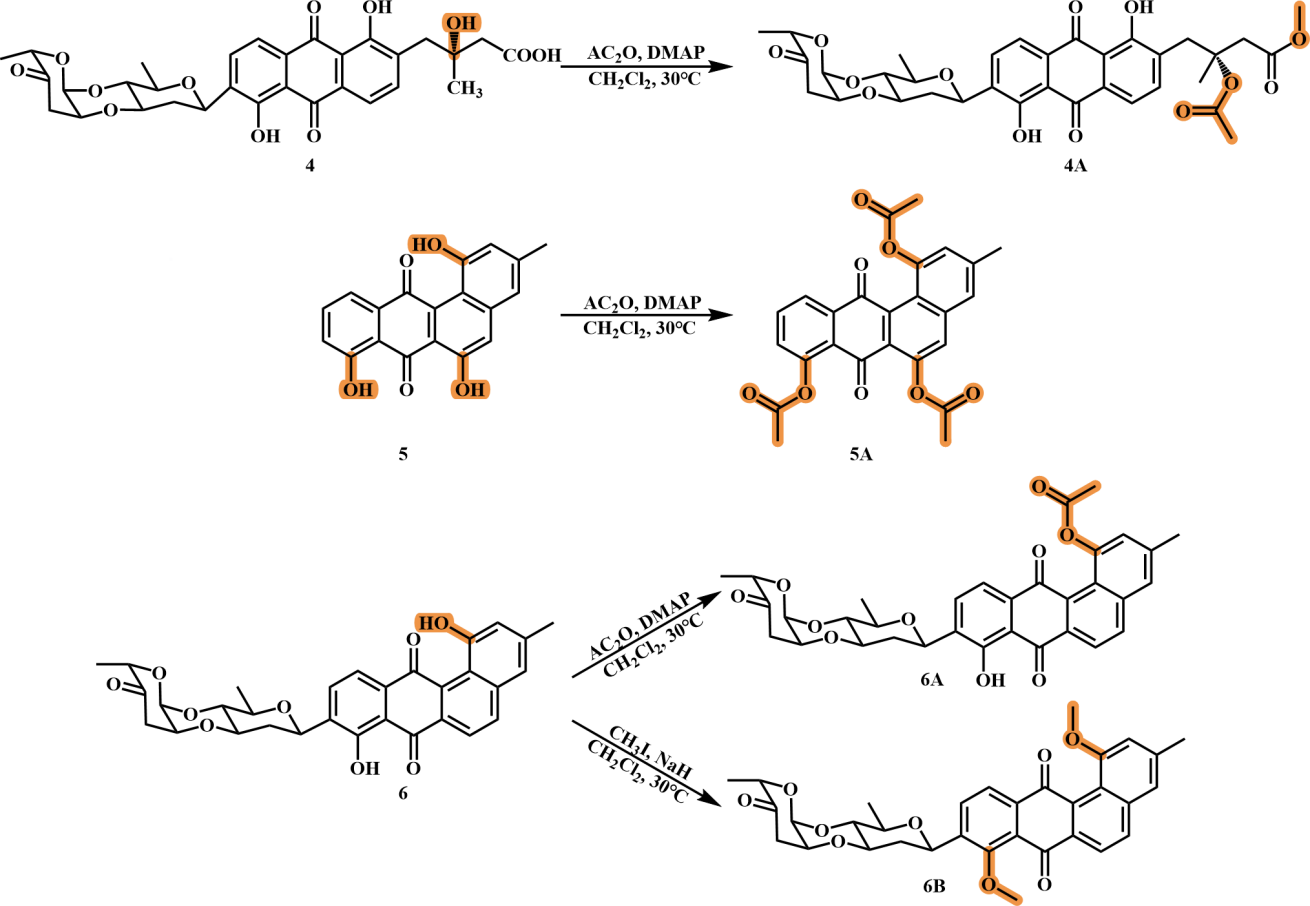
Derivatization pathways of compounds **4**, **5**, and **6**.

**Fig 6:**
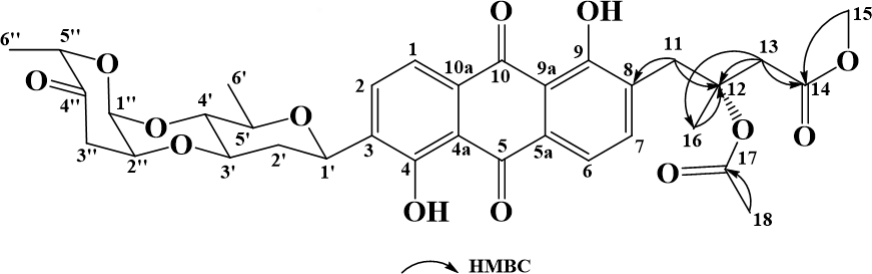
Key HMBCs (arrows) of compound **4A**.

Due to the high yield of compounds **4–6** (approximately 150 mg) in BYF-106, further derivatization structural modifications were carried out on compounds **4–6** (Fig. 5). Hydroxyacetylation reactions were performed on compounds **4** and **5**, resulting in derivatives **4A** and **5A**. Compound **6** was subjected to hydroxyacetylation and hydroxymethylation reactions, resulting in derivatives **6A** and **6B**. The specific results were as follows:

Compound **4A** was obtained as a yellow solid. Its molecular formula was determined as C_34_H_36_O_13_ by HR-ESI-MS *m*/*z* 651.2070 [M-H]^-^ (calcd for C_34_H_35_O_13_^-^, 651.2078). The structure of compound **4A** was determined by comparison with compound **4** and the experiments of 2D NMR (HSQC, HMBC, and ^1^H-^1^H COSY). The ^1^H NMR spectrum showed two -OH signals *δ*_H_ [12.94 (1H, s), 13.30 (1H, s)], a methoxy hydrogen signal at *δ*_H_ 3.60 (3H, s), four methyl hydrogen signals at *δ*_H_ [1.95 (3H, s), 1.42 (3H, s), 1.25 (3H, d, *J* = 6.0 Hz), and 1.24 (3H, s)]. ^13^C NMR and DEPT135 spectrum showed thirty-four carbonyl carbon signals: twelve aromatic carbon signals (*δ*_C_ 118.9, 133.6, 137.0, 158.0, 115.3, 131.6, 118.4, 139., 133.1, 169.7, 115.3, and 131.8), four carbonyl groups signals (*δ*_C_ 187.7, 169.7, 169.9, 208.5), a methoxy carbon signal *δ*_C_ 51.3, two methylene carbon signals (*δ*_C_ 36.6, 41.8), four methyl carbon signals (*δ_C_* 16.0, 17.3, 22.0, and 23.7). These data indicated the presence of an additional acetyl group and a methoxy group in the structure of compound **4A**. The formation of the methoxy group may be attributed to the carboxylic acid group of **4** with acetic acid to form an acetic anhydride during the reaction process. Subsequently, methylation occurred during the dissolution process with methanol solution due to the instability of the acetic anhydride. HMBC spectrum showed the correlations from the H-13 (*δ*_H_ 3.11, 2.88) and H-15 (*δ*_H_ 3.60) to C-14 (*δ*_C_ 169.7), indicating the presence of the methoxy group at C-14 (Fig. 6, Fig. S13 to S19). Meanwhile, compound **4A** was in the absence of two –OH signals (12-OH, 14-OH) compared with compound **4**. Thus, an acetyl group and an oxygen methyl group were located at C-12 and C-14 of compound **4A**, respectively.

Compound **5A** was obtained as a yellow solid. Its molecular formula was determined as C_25_H_18_O_8_ by HR-ESI-MS *m*/*z* 469.0891 [M + Na]^+^ (calcd for C_25_H_18_O_8_Na^+^, 469.0899). The structure of compound **5A** was determined by comparison with compound **5**. The ^1^H NMR spectrum of **5A** showed six aromatic hydrogen atom signals *δ*_H_ [77.91 (1H, d, *J* = 7.5 Hz), 7.75(1H, t, *J* = 8.5 Hz), 7.65 (1H, s), 7.49 (1H, s), 7.37 (1H, d, *J* = 7.9 Hz), 7.31 (1H, s)], four methyl group signals *δ*_H_ [2.55 (3H, s), 2.46 (3H, d, *J* = 1.5 Hz), 2.44 (3H, d, *J* = 1.4 Hz), and 2.36 (3H, d, *J* = 1.4 Hz)]. The absence of three hydroxyl signals (1-OH, 6-OH, and 8-OH) in the ^1^H NMR spectrum of compound **5A** indicated acetylation at these positions. ^13^C NMR spectrum showed seventeen aromatic carbon signals (*δ*_C_ 149.6, 147.2, 145.8, 141.2, 138.0, 137.0, 136.4, 134.8, 129.1, 127.2, 127.0, 125.8, 125.1, 125.0, 123.8, 120.0, and 110.2), five carbonyl group signals (*δ*_C_ 185.2, 180.6, 169.9, 169.3, and 168.5), four methyl carbon signals (*δ*_C_ 21.6, 21.2, 21.2, and 21.2) (Fig. S20 to S22). These data also indicated the acetyl groups were located at C-1, C-6, and C-8.

Compound **6A** was obtained as a yellow solid. Its molecular formula was determined as C_33_H_30_O_10_ by HR-ESI-MS 587.1908 [M + H]^+^ (calcd for C_33_H_31_O_10_^+^, 587.1917). The structure of compound **6A** was determined by comparison with compound **6**. The ^1^H NMR spectrum of **6A** showed a –OH signal *δ*_H_ 12.65 (1H, s), six aromatic hydrogen atoms signals *δ*_H_ [8.26 (1H, d, *J* = 8.3 Hz), 8.06 (1H, t, *J* = 8.5 Hz), 7.89 (1H, d, *J* = 7.6 Hz), 7.59 (2H, m), and 7.34 (1H, s)], four methyl groups signals *δ*_H_ [1.39 (3H, m), 1.42 (3H, m), 2.33(3H, s), and 2.57 (3H, s)]. Compound **6A** was in the absence of a –OH signal (1-OH) compared with compound **6**, indicating that acetylation may occur at positions C-1. The ^13^C NMR spectrum showed fifteen aromatic carbon signals (*δ*_C_ 158.1, 147.5, 140.6, 138.6, 136.0, 135.1, 133.9, 133.8, 133.7, 126.2, 125.9, 122.0, 121.7, 118.5, and 115.0), five carbonyl group signals (*δ*_C_ 207.7, 188.7, 184.9, and 168.9), four methyl carbon signals (*δ_C_* 21.7, 21.1, 16.3, and 17.7) (Fig. S23 to S25). These data also indicated the presence of an acetyl group at C-1.

Compound **6B** was obtained as a yellow solid. Its molecular formula was determined as C_33_H_32_O_9_ by HR-ESI-MS 573.2115 [M + H]^+^ (calcd for C_33_H_33_O_9_^+^, 573.2124). The structure of compound **6B** was determined by comparison with compound **6**. The ^1^H NMR spectrum of **6B** showed six aromatic hydrogen atom signals *δ*_H_ [8.20 (1H, d, *J* = 8.4 Hz), 7.97 (1H, d, *J* = 7.6 Hz), 7.89 (2H, m), 7.26 (1H, m), 6.90 (1H, s)], five methyl signals *δ_H_* [3.97 (6H, s), 2.54 (3H, s), 1.26 (6H, m)]. Compound **6B** was in the absence of two –OH signals (1-OH and 8-OH) compared with compound **6**, indicating that acetylation may occur at positions C-1 and C-8. The ^13^C NMR spectrum showed sixteen aromatic carbon signals (*δ*_C_ 157.6, 157.4, 156.8, 140.8, 140.7, 138.4, 138.2, 138.1, 133.9, 133.9, 132.7, 132.6, 122.6, 122.5, 120.2, 111.5), three carbonyl group signals (*δ*_C_ 207.6, 185.7, 182.3), three methyl carbon signals (*δ*_C_ 22.3, 16.3, 17.7), and two methoxy carbon signals (*δ*_C_ 56.3, 62.8) (Fig. S26 to S28). These data also indicated the presence of the acetyl groups at C-1 and C-8.

### Antibacterial assay

The disc diameters of the zone of inhibition (ZOI) of compounds **1**, **3–10**, **4A–6A,** and **6B** against two gram-positive bacteria (*S. aureus*, MRSA) and three gram-negative bacteria (Psa, Xoo, and Xoc) are shown in Table 3. The results showed that compounds **3–5** displayed strong antibacterial activities against MRSA with ZOI values of 12.33, 11.83, and 11.33 mm, respectively, which were weaker than levofloxacin with a ZOI value of 17.17 mm. Compound **3** also exhibited significant antibacterial activity against *S. aureus* and Psa with ZOI values of 13.67 and 17.67 mm, respectively, which was weaker than levofloxacin (ZOI = 35.67 and 32.33 mm) and gentamicin sulfate (ZOI = 20.67 and 24.50 mm). In addition, compounds **4** and **5** exhibited moderate antibacterial effects against *S. aureus* with ZOI values of 13.17 mm and 11.20 mm, respectively. Simultaneously, compounds **4** and **5** also exhibited moderate antibacterial effects against Psa with ZOI values of 12.67 mm and 11.50 mm, respectively.

**TABLE 3:** Zone of inhibition (ZOI, mm) of **1**, **3–10**, **4A–6A,** and **6B** against pathogenic bacteria^*c*^

Compound	*S. aureus*	MRSA	Psa	Xoo	Xoc
1	NI^*a*^	NI	NI	NI	NI
3	13.67 ± 0.29	12.33 ± 0.76	17.67 ± 0.29	NI	NI
4	13.17 ± 0.76	11.83 ± 0.58	12.67 ± 0.76	NI	NI
5	11.20 ± 0.50	11.33 ± 0.58	11.50 ± 0.50	NI	NI
6	NI	NI	6.83 ± 0.29	NI	NI
7	NI	NI	8.83 ± 0.29	NI	NI
8	NI	NI	NI	NI	NI
9	7.67 ± 1.04	NI	NI	NI	NI
10	NI	NI	NI	NI	NI
4A	NI	NI	NI	NI	NI
5A	NI	NI	10.67 ± 0.58	NI	NI
6A	NI	NI	NI	NI	NI
6B	NI	NI	NI	NI	NI
Gentamicin sulfate^*b*^	20.67 ± 1.76	NI	24.50 ± 0.50	21.50 ± 0.00	15.33 ± 0.76
Levofloxacin^*b*^	35.67 ± 0.58	17.17 ± 1.76	32.33 ± 1.04	41.67 ± 0.29	39.17 ± 1.76

^*a*^ NI means not inhibited.

^*b*^ Positive control; the concentration for the test is 50 µg/filter paper.

^*c*^ Psa: *Pseudomonas syringae* pv. *actinidae*; Xoo: *Xanthomonas oryzae* pv. *oryzae*; Xoc: *Xanthomonas oryzae* pv. *oryzicola*.

### NO inhibition assay

The inhibitory effects of tested compounds (**1**, **3–10**, **4A-6A,** and **6B**) on NO generation in LPS stimulated in RAW264.7 cells were evaluated. The results showed no cytotoxic effects for tested metabolites (except **5**, **5A,** and **6B**) were observed at 50 µM. As shown in Table 4, compound **1** displayed a strong inhibitory effect on NO production, with an IC_50_ value of 4.8 µM, which was comparable to that of Bay11-7082 with an IC_50_ value of 2.1 µM. Simultaneously, compounds **4, 7,** and **6A** exhibited significant inhibitory effects of NO production, with IC_50_ values of 10.7–16.2 µM. In addition, compounds **8** and **9** exhibited mild and medium inhibitory effects of NO production, with IC_50_ values of 40.2 and 22.7 µM.

**TABLE 4:** Inhibitory effects of **1**, **3**, **4**, **6–10**, **4A,** and **6A** on LPS-induced NO generation in RAW264.7 cells

Compound	NO inhibition IC_50_ (μM)
1	4.8 ± 0.9
3	>50
4	16.2 ± 3.8
6	>50
7	13.1 ± 2.3
8	40.2 ± 5.1
9	22.7 ± 5.4
10	>50
4A	>50
6A	10.7 ± 1.4
Bay11-7082^*a*^	2.1 ± 0.7

^*a*^ Positive control.

To further investigate the inhibitory effect of compound **1** on NO generation, the possible binding mode between compound **1** and the COX-2 protein was predicted by molecular docking. The results indicated compound **1** was well docked into the active site of COX-2 protein, with a binding energy of −42.35 Kcal/mol. As shown in Fig. S29, the indole ring of compound **1** formed a hydrophobic interaction with Arg202. The lactone ring could form an electrostatic interaction with Arg202. The hydroxyl group at C-12b formed a hydrogen bond with ASN130. The carbonyl group at C-1′′′ formed a hydrogen bond with Arg202. The methyl group at C-6″ formed a hydrophobic interaction with LYS201. This investigation may provide a rationalized anti-inflammatory mechanism of compound **1**.

### Cytotoxicity assay

The tested compounds (**1**, **3–10**, **4A**–**6A,** and **6B**) were evaluated for cytotoxicity against HCT-116, HT-29, and A375. As shown in Table 5, most of the tested compounds exhibited significant cytotoxicity against HCT-116, HT-29, and A375. Compound **5A** showed extremely strong cytotoxic activity against A375 (IC_50_ <0.2 µM), which was stronger than SEL120-34A (IC_50_ = 5.9 µM). Compounds **1**, **5,** and **9** exhibited strong cytotoxic activities against A375, with IC_50_ values of 13.6, 16.1, and 7.6 µM, respectively. Meanwhile, Compounds **4**, **8**, **6A,** and **6B** displayed medium cytotoxic activities against A375, with IC_50_ values of 32.3–55.4 µM. Compounds **5** and **5A** showed extremely strong cytotoxic activities against HCT-116, with IC_50_ values of 9.8 and 2.2 µM, respectively, which were stronger than SEL120-34A with an IC_50_ value of 11.3 µM. Compounds **1** and **9** showed strong cytotoxic activities against HCT-116, which were comparable to that of SEL120-34A. Compounds **4**, **8**, **6A**, **6B,** and **10** displayed mild to medium cytotoxic activities against HCT-116, with IC_50_ values of 29.3–77.1 μM. In addition, compounds **5**, **7**, **10**, and **5A** displayed strong cytotoxic activities against HT-29, with IC_50_ values of 9.5–17.2 µM, which is stronger than SEL120-34A (IC_50_ = 33.8 µM). Compounds **1**, **4**, **6B**, and **9** showed mild to medium cytotoxic activities against HT-29, with IC_50_ values of 44.3–88.7 µM.

**TABLE 5:** Cytotoxic effects of **1**, **3–10**, **4A–6A,** and **6B** against HCT-116, HT-29, and A375 cells (IC_50_, μM)^*a*^

Compound	HCT-116	HT-29	A375
1	11.7 ± 1.2	67.1 ± 3.2	13.6 ± 1.4
3	>100	>100	>100
4	41.0 ± 3.1	88.7 ± 3.0	32.2 ± 1.1
5	9.8 ± 1.4	17.2 ± 0.8	16.1 ± 2.1
6	>100	>100	>100
7	>100	8.6 ± 0.8	>100
8	39.4 ± 3.9	>100	33.5 ± 2.6
9	18.3 ± 1.2	44.3 ± 3.9	7.6 ± 1.4
10	77.1 ± 4.9	9.5 ± 0.9	>100
4A	>100	>100	>100
5A	2.2 ± 0.7	9.9 ± 1.2	< 0.2
6A	35.0 ± 3.3	>100	55.4 ± 2.9
6B	29.3 ± 3.9	82.9 ± 3.5	37.5 ± 1.9
SEL120-34A^*b*^	11.3 ± 1.9	33.8 ± 1.4	5.9 ± 1.43

^*a*^ HCT-116, HT-29: human colon cancer cell lines; A375: human malignant melanoma cell lines.

^*b*^ Positive control.

To further explore the cytotoxicity of compound **5A** against A375, the possible binding mode between compound **5A** and the BRAF^V600E^ protein was predicted by molecular docking. The results indicated compound **5A** was well docked into the active site of BRAF^V600E^ protein, with a binding energy of −73.10 Kcal/mol. As shown in Fig. S30, the benzene ring of compound **5A** formed hydrophobic interactions with TRP531 and ILE463, respectively. The carbonyl group at C-16 formed a hydrogen bond with GLY464. The carbonyl group at C-18 formed a hydrogen bond with ILE463. The results of molecular docking may indicate a rationalized cytotoxicity mechanism of compound **5A** against A375.

## DISCUSSION

Bioassay and MS-guided separation of the EtOAc extract from *S. lannensis* BYF-106 afforded 10 C-glycoside angucycline-related analogs. The C-glycoside angucycline family of natural products has been one of the hotspots of drug research due to its unique structures and diverse bioactivities. In this study, partial C-glycoside angucycline-related analogs, vineomycinone B2 (**3**), fridamycin D (**4**), and 6-hydroxytetrangulol (**5**), displayed significant antibacterial activities against two foodborne pathogens (*S. aureus*, methicillin-resistant *S. aureus*) and a plant pathogen (*P. syringae* pv. *actinidae*), indicating their potential as antibacterial agents in food safety and agriculture. In addition, our research first revealed the significant anti-inflammatory activities of C-glycoside angucycline-related analogs. Among these metabolites, compound **1** displayed a strong inhibitory effect on NO production (IC_50_ = 4.8 µM). Moreover, *S. lannensis* previously was reported to produce a well-known antibiotic (actinomycin D) (39). To our knowledge, it was the first report of two new C-glycoside angucycline-related analogs (**1** and **2**) and nine known metabolites (**3–10**) produced by *S. lannensis* 106 isolated from the surface of *O. formosanus*.

Compound **4** displayed broad-spectrum biological activities, whereas compound **3** only showed significant antibacterial activities against *S. aureus*, MRSA, and Psa. Structurally, this difference may be attributed to the presence of the *α*-cinerulose B-(1→4, 2→3)-*β*-olivosyl moiety at C-3 in compound **4**. Furthermore, the acetylation product **4A** did not show any marked antibacterial, anti-inflammatory, and cytotoxic activities, indicating the hydroxyl and carboxyl groups on the lipid chain of compound **4** may also be the main bioactive groups. Compound **5** exhibited broad-spectrum antibacterial and cytotoxic activities. Intriguingly, the acetylation product **5A** exhibited extremely strong cytotoxic activity, although it was inactive against the tested pathogenic bacteria. These results indicated the hydroxyl groups at C-1, C-6, and C-8 in compound **5** may be the main antibacterial active groups. Meanwhile, acetyl groups at C-1, C-6, and C-8 could increase the cytotoxic activity of compound **5**. Compound **6** did not present any bioactivities, while the acetylation and methylation products (**6A** and **6B**) showed significant cytotoxic activity. In addition, the acetylation product **6A** also presented a strong inhibitory effect on NO production (IC_50_ = 10.7 µM). Comparing the structures of compounds **6**, **6A,** and **6B**, the substitute of the acetyl group at C-1 in **6** could enhance anti-inflammatory and cytotoxic activities.

Finally, the potential mechanisms of action of compounds **1** and **5A** were explored through molecular docking analysis. COX-2 is a pro-inflammatory enzyme that has been reported to play a crucial role in the inflammatory process (40). The results of molecular docking demonstrated the indole ring and lactone ring of compound **1** formed a hydrophobic and electrostatic interaction with Arg202 of Cox-2, respectively. Thus, it could be speculated that the unique indole-pyranone of compound **1** may improve anti-inflammatory activity compared to other C-glycoside angucycline-related analogs. It has been reported that approximately 50% of melanomas are associated with activation mutations in the BRAF gene. The most common BRAF mutation is the BRAF^V600E^ activating mutation (41). The research of molecular docking between compound **5A** and BRAF^V600E^ showed the carbonyl groups at C-16 and C-18 could form hydrogen bonds with GLY464 and ILE463, respectively, which further indicated that acetyl groups could increase the cytotoxic activity of compound **5A**.

### Conclusions

In summary, two novel C-glycoside angucycline-related analogs (**1** and **2**) along with eight known metabolites (**3–10**) were isolated and characterized from *S. lannensis* BYF-106. Furthermore, four new derivative compounds (**4A**, **5A**, **6A,** and **6B**) were synthesized through acetylation and methylation based on compounds **4**, **5,** and **6**. Among the tested compounds, vineomycinone B2 (**3**), fridamycin D (**4**), and 6-hydroxytetrangulol (**5**) exhibited broad-spectrum antibacterial activities against *S. aureus*, MRSA, and *P. syringae* pv. *actinidae*. In addition, urdamycin Y (**1**) strongly inhibited NO production in LPS-induced RAW264.7 cells, and its potential anti-inflammatory mechanism was investigated through molecular docking simulations. Most of the tested metabolites showed significant cytotoxic activities against HCT-116, HT-29, and A375, with the acetyl derivative (**5A**) exhibiting exceptionally potent cytotoxic effects against A375. The potential cytotoxic activity mechanism of **5A** against A375 was also proposed by molecular docking. Overall, these findings provide a theoretical basis for the development of new bioactive drugs in food, agriculture, and biomedical industries.

## Data Availability

The NMR data presented in this study have been deposited into Zenodo at https://doi.org/10.5281/zenodo.15018418. The MS data can be found and accessed at http://gnps.ucsd.edu/ProteoSAFe/status.jsp?task=140e26e38cbd40c089fcebdb23630aa5.

## SUPPLEMENTAL MATERIAL

Supplemental material spectrum.01818-24-s0001.docx Fig. S1 to S30; Table S1.

ASM does not own the copyrights to Supplemental Material that may be linked to, or accessed through, an article. The authors have granted ASM a non-exclusive, world-wide license to publish the Supplemental Material files. Please contact the corresponding author directly for reuse.
