# Supplementary material for: Structural characterization, derivatization, and bioactivities of secondary metabolites produced by termite-associated Streptomyces lannensis BYF-106
Source: Microbiol Spectr. 2025 Apr 15;13(5):e01818-24. doi: 10.1128/spectrum.01818-24 (PMC12054111; doi:10.1128/spectrum.01818-24)
Supplement: Supplemental material — Fig. S1 to S30; Table S1. [file spectrum.01818-24-s0001.docx]

**Supplementary Information**

**Structural Characterization, Derivatization and Bioactivities of Secondary Metabolites Produced by Termite-associated *Streptomyces lannensis* BYF-106**

Jun Wu^1, 2^, Miao Zhang^1^, Jian Tao^1^, MengRu, Liu^1^, JianHao Xiong^1^, TaoShan Jiang^1^, YaXuan Wang^1^, XiaoHong Li^1^, YueYue Li^1^, CaiPing Yin^1^, ShuXiang Zhang^1^, XinHua Liu^3^, YingLao Zhang^1^,*

^1^School of Life Science, Anhui Agricultural University, 130 West Changjiang Rd., Hefei 230036 Anhui, China

^2^Center for Biological Science and Technology, Advanced Institute of Natural Sciences, Beijing Normal University, Zhuhai, Guangdong 519087, China

^3^School of Pharmacy, Anhui Medical University, 81 Meishan Rd., Hefei 230036 Anhui, China
Yinglao Zhang: zhangyl@ahau.edu.cn; Tel.: +86-0551-6578-0690.

*Corresponding author

**Supporting information description**

Table S1. Deduced functions of ORFs in *UDM* biosynthesis gene cluster of *S*. *lannensis* BYF-106.

Figure S1. ^1^H NMR spectrum of urdamycin Y (**1**) at 600 MHz in actone-*d_6_*.

Figure S2. ^13^C NMR spectrum of urdamycin Y (**1**) at 150 MHz in actone-*d_6_*.

Figure S3. DEPT135 spectrum of urdamycin Y (**1**) at 150 MHz in actone-*d_6_*_._

Figure S4. HSQC spectrum of urdamycin Y (**1**) in actone-*d_6_*_._

Figure S5. HMBC spectrum of urdamycin Y (**1**) in actone-*d_6_*_._

Figure S6. ^1^H-^1^H COSY spectrum of urdamycin Y (**1**) in actone-*d_6_*_._

Figure S7. HR-ESI-MS spectrum of urdamycin Y (**1**).

Figure S8. UV spectrum of urdamycin Y (**1**) in MeOH.

Figure S9. IR spectrum of urdamycin Y (**1**).

Figure S10. ^1^H NMR spectrum of grincamycin W (**2**) at 600 MHz in actone-*d_6_*.

Figure S11. ^13^C NMR spectrum of grincamycin W (**2**) at 150 MHz in actone-*d_6_*.

Figure S12. HR-ESI-MS spectrum of grincamycin W (**2**).

Figure S13. ^1^H NMR spectrum of **4A** at 600 MHz in DMSO-*d_6_*.

Figure S14. ^13^C NMR spectrum of **4A** at 150 MHz in DMSO-*d_6_*.

Figure S15. DEPT135 spectrum of **4A** at 150 MHz in DMSO-*d_6_*.

Figure S16. HSQC spectrum of **4A** in DMSO-*d_6_*_._

Figure S17. HMBC spectrum of **4A** in DMSO-*d_6_*_._

Figure S18. ^1^H-^1^H COSY spectrum of **4A** in DMSO-*d_6_*_._

Figure S19. HR-ESI-MS spectrum of **4A**.

Figure S20. ^1^H NMR spectrum of **5A** at 600 MHz in CDCl_3_.

Figure S21. ^13^C NMR spectrum of **5A** at 150 MHz in CDCl_3_.

Figure S22. HR-ESI-MS spectrum of **5A**.

Figure S23. ^1^H NMR spectrum of **6A** at 600 MHz in CDCl_3_.

Figure S24. ^13^C NMR spectrum of **6A** at 150 MHz in CDCl_3_.

Figure S25. HR-ESI-MS spectrum of **6A**.

Figure S26. ^1^H NMR spectrum of **6B** at 600 MHz in CDCl_3_.

Figure S27. ^13^C NMR spectrum of **6B** at 150 MHz in CDCl_3_.

Figure S28. HR-ESI-MS spectrum of **6B**.

Figure S29. Molecular docking model of **1** into COX-2 binding site.

Figure S30. Molecular docking model of **5A** into BRAF^V600E^ binding site.

**Table S1**. Deduced functions of ORFs in *UDM* biosynthesis gene cluster of *S. lannensis* BYF-106.

| CDS | Size (aa) | Putative function | Homologous protein (origin) | Identify |
| --- | --- | --- | --- | --- |
| 50 | 364 | Ferredoxin oxidoreductase | SqnU (*Streptomyces* sp. KY 40-1) | 97.76% |
| 51 | 639 | Ferredoxin oxidoreductase | SqnT (*Streptomyces* sp. KY 40-1) | 98.19% |
| 67 | 378 | Glycosyltransferase | SqnG3 (*Streptomyces* sp. KY 40-1) | 97.07% |
| 69 | 404 | Glycosyltransferase | SqnG2 (*Streptomyces* sp. KY 40-1) | 96.78% |
| 70 | 430 | Glycosyltransferase | SqnG1 (*Streptomyces* sp. KY 40-1) | 97.91% |
| 72 | 665 | Monooxygenase | SqnM (*Streptomyces* sp. KY 40-1) | 95.94% |
| 73 | 311 | Cyclase | SqnL (*Streptomyces* sp. KY 40-1) | 96.78% |
| 74 | 261 | Ketoreductase | SqnK (*Streptomyces* sp. KY 40-1) | 99.62% |
| 75 | 89 | Acyl carrier protein | SqnJ (*Streptomyces* sp. KY 40-1) | 95.51% |
| 76 | 408 | Ketoacyl synthase CLF | SqnI (*Streptomyces* sp. KY 40-1) | 98.04% |
| 77 | 417 | Ketoacyl synthase alpha subunit | SqnH (*Streptomyces* sp. KY 40-1) | 98.80% |
| 78 | 108 | Cyclase | SqnBB (*Streptomyces* sp. KY 40-1) | 98.15% |
| 79 | 495 | Monooxygenase | SqnF (*Streptomyces* sp. KY 40-1) | 97.78% |
| 82 | 199 | Reductase | SqnC (*Streptomyces* sp. KY 40-1) | 98.49% |
| 84 | 372 | NADH oxidase | MBT84-08735 (*Streptomyces* sp. MBT84) | 98.39% |


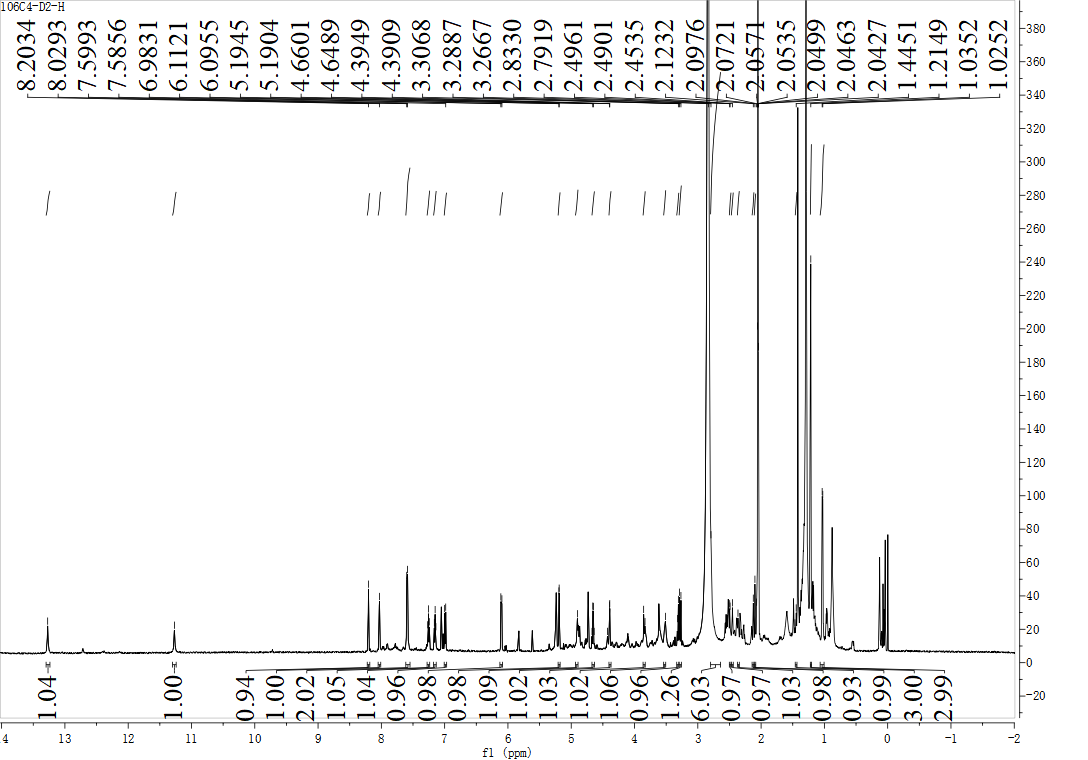


**Figure S1**. ^1^H NMR spectrum of urdamycin Y (**1**) at 600 MHz in actone-*d_6_*.


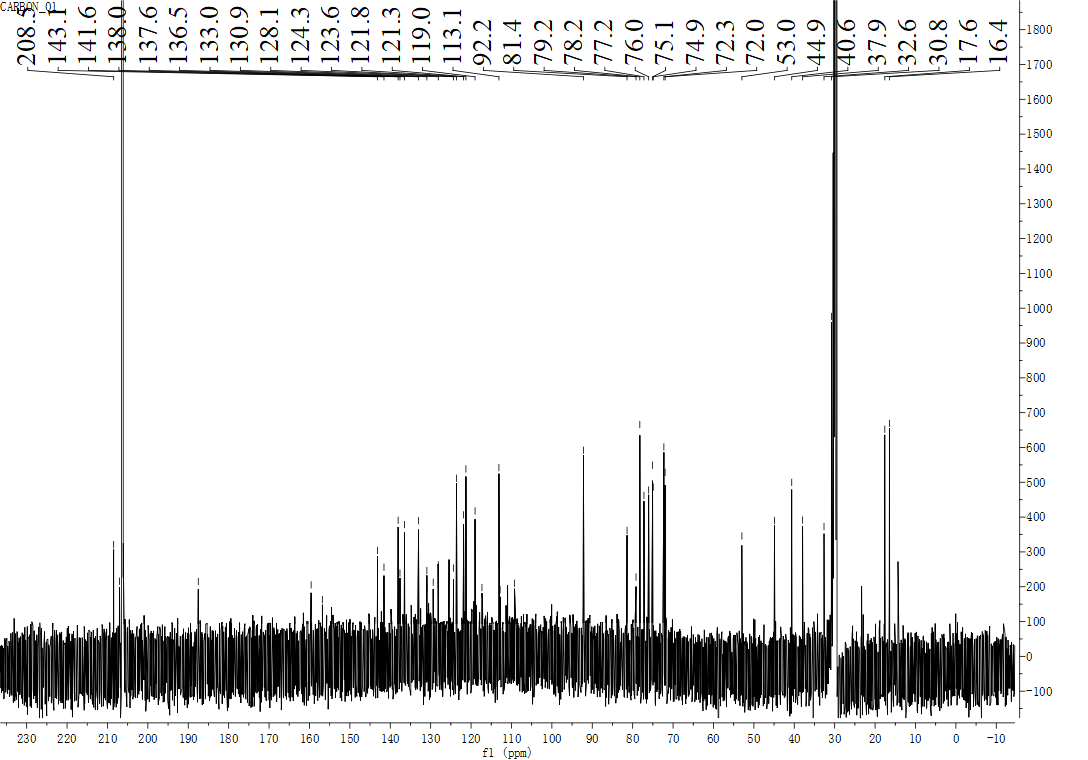


**Figure S2**. ^13^C NMR spectrum of urdamycin Y (**1**) at 150 MHz in actone-*d_6_*.


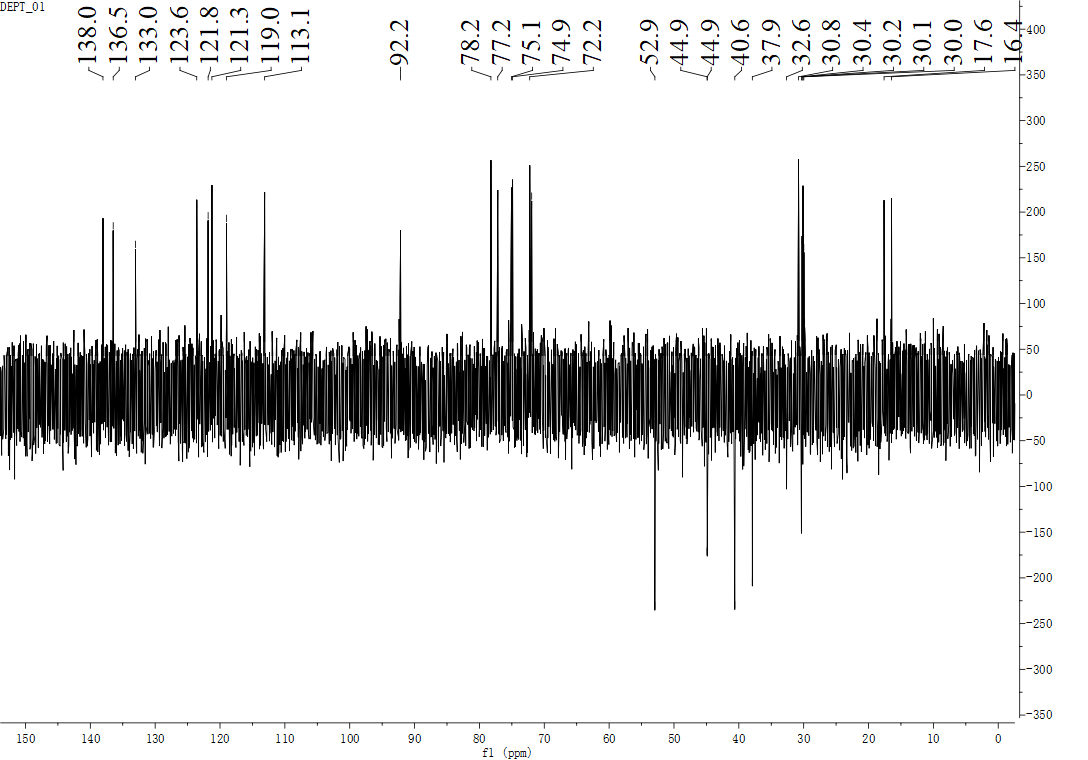


**Figure S3**. DEPT135 spectrum of urdamycin Y (**1**) at 150 MHz in actone-*d_6_*_._


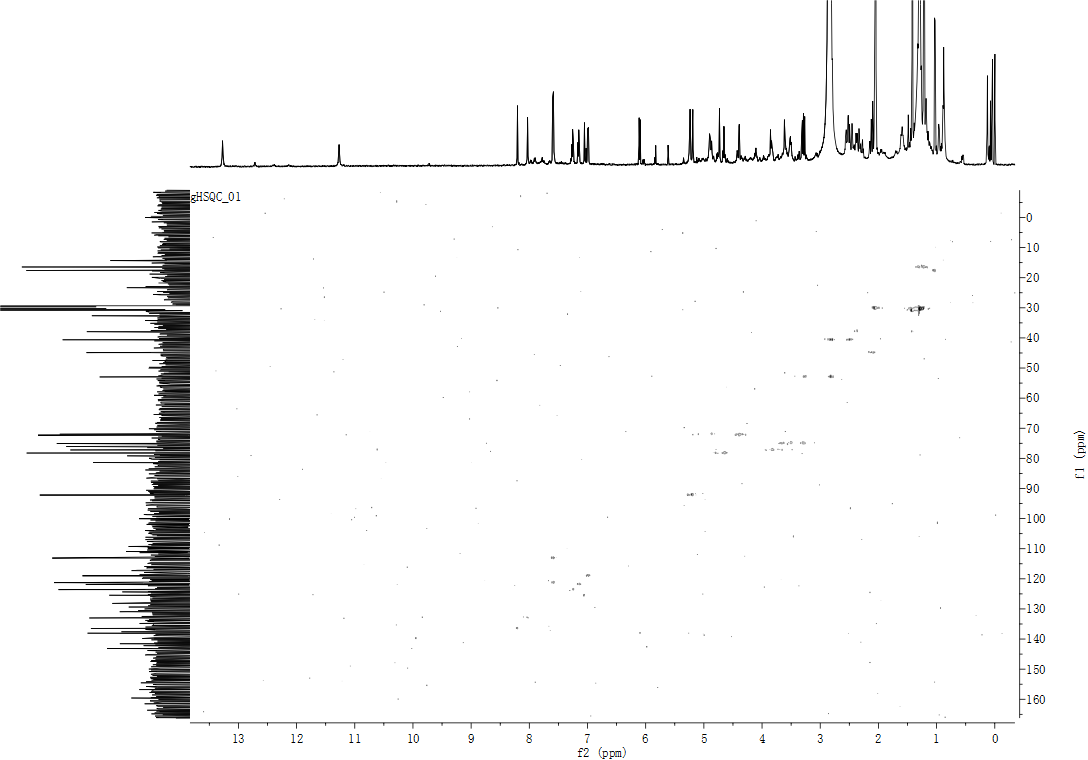


**Figure S4**. HSQC spectrum of urdamycin Y (**1**) in actone-*d_6_*_._


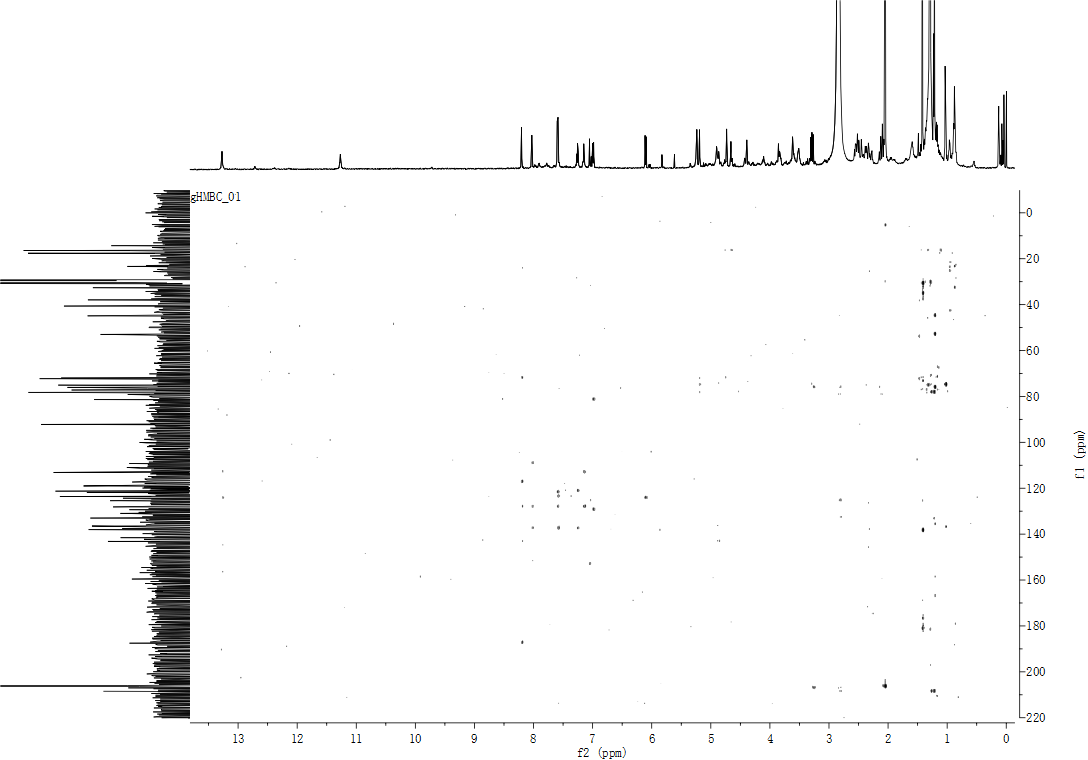


**Figure S5**. HMBC spectrum of urdamycin Y (**1**) in actone-*d_6_*_._


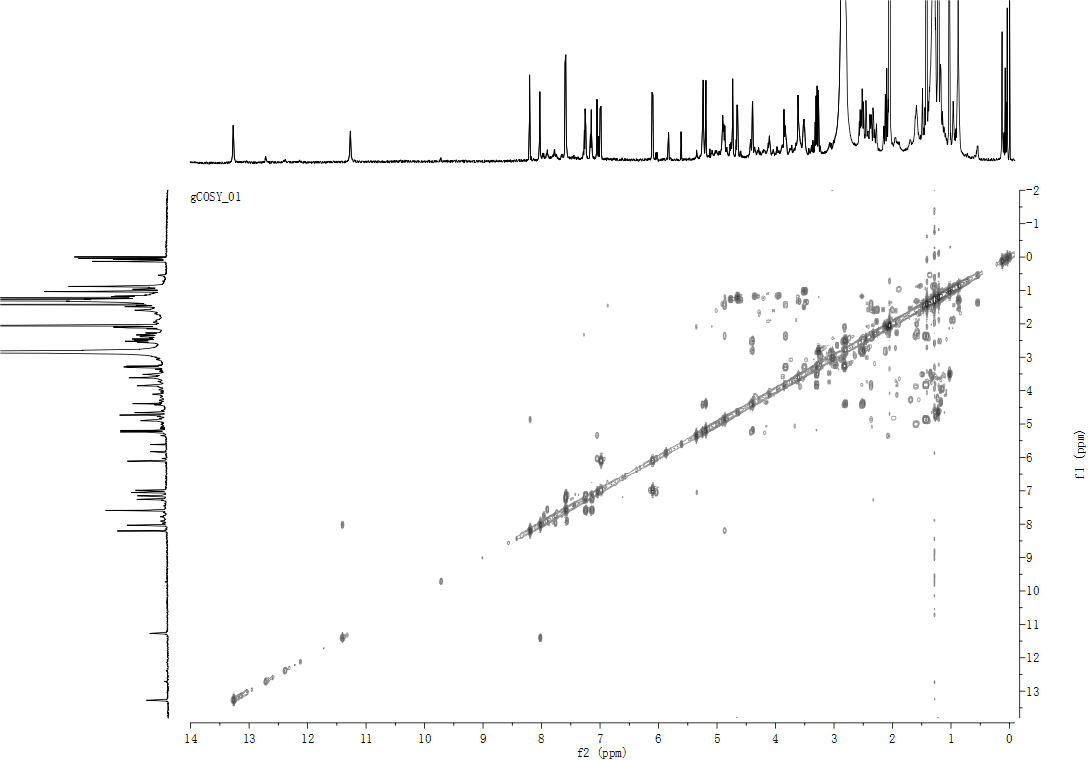


**Figure S6**. ^1^H-^1^H COSY spectrum of urdamycin Y (**1**) in actone-*d_6_*_._


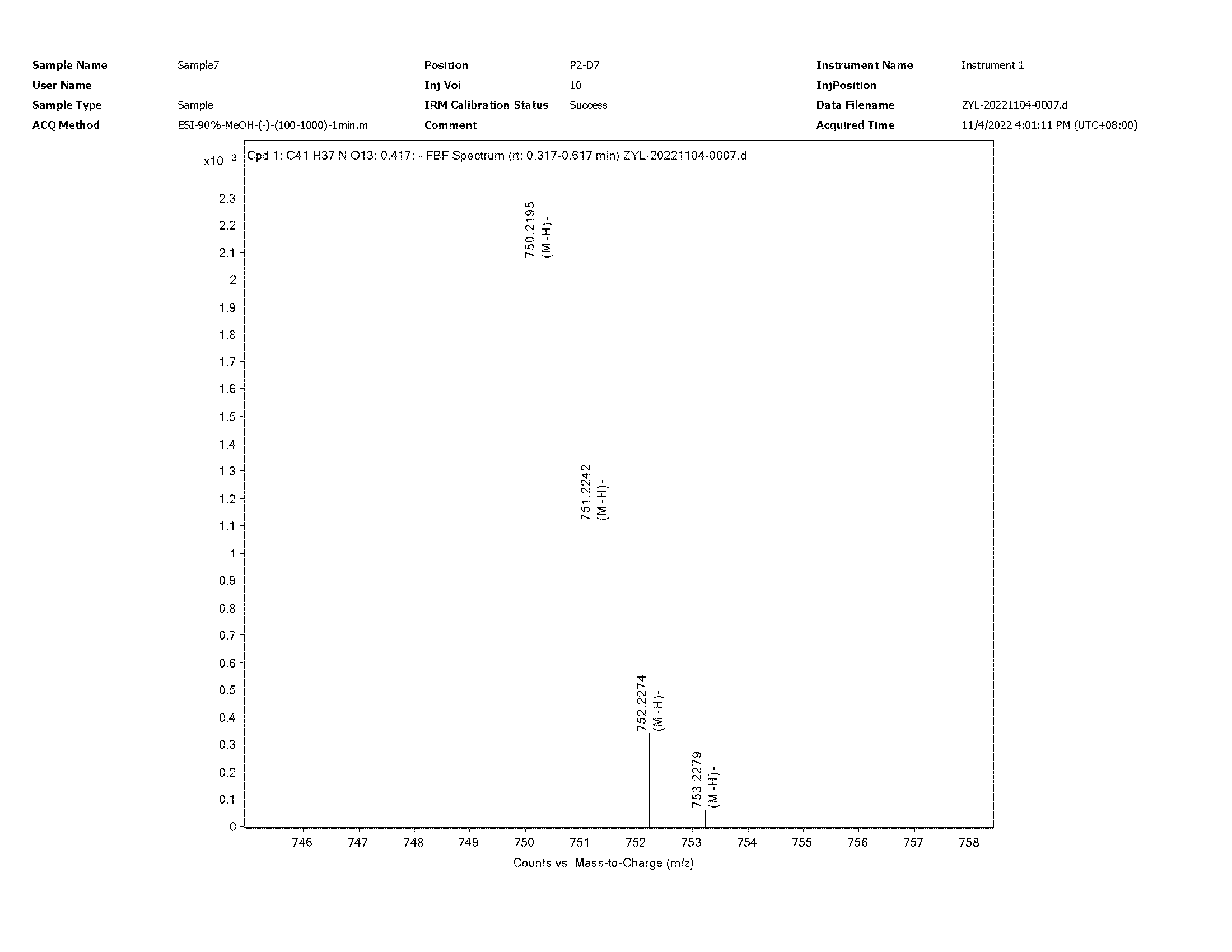


**Figure S7**. HR-ESI-MS spectrum of urdamycin Y (**1**).


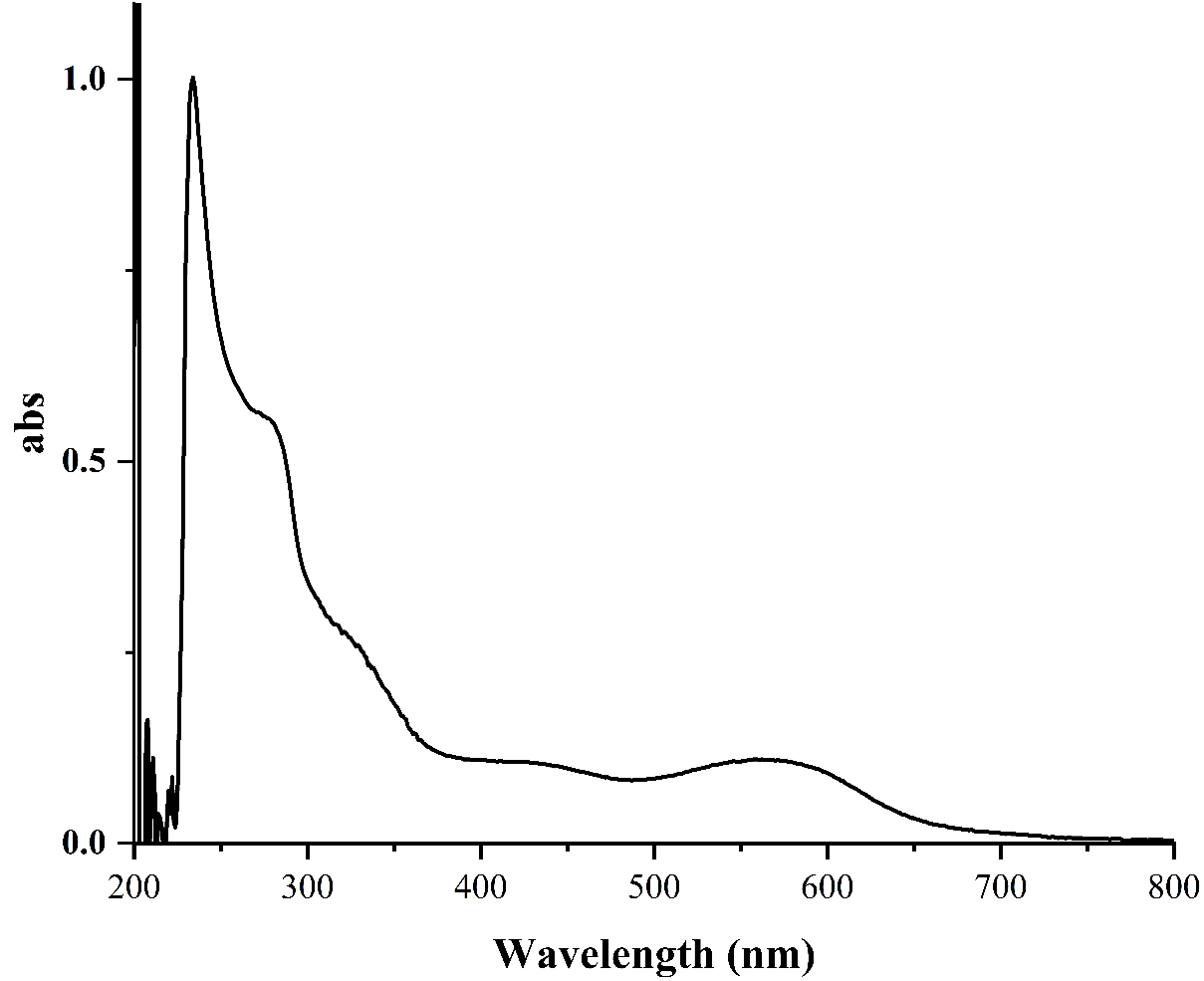


**Figure S8**. UV spectrum of urdamycin Y (**1**) in MeOH.


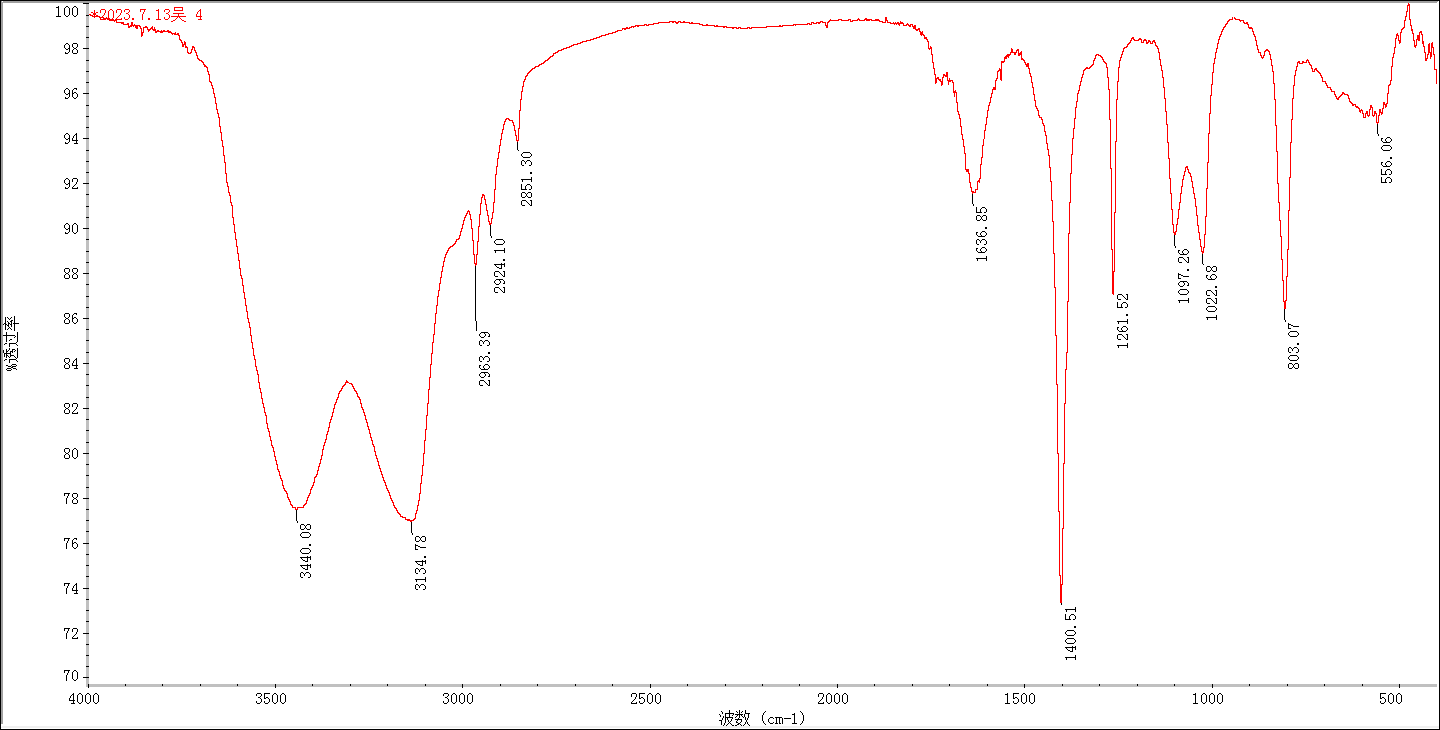


**Figure S9**. IR spectrum of urdamycin Y (**1**).


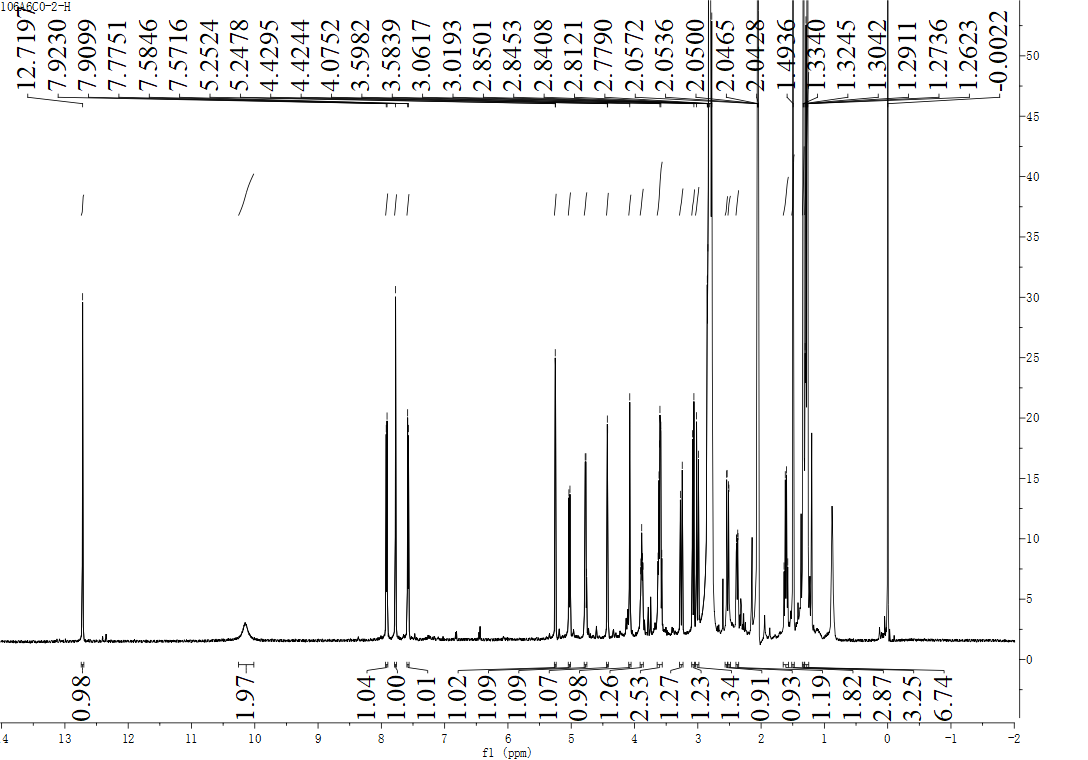


**Figure S10**. ^1^H NMR spectrum of grincamycin W (**2**) at 600 MHz in actone-*d_6_*.


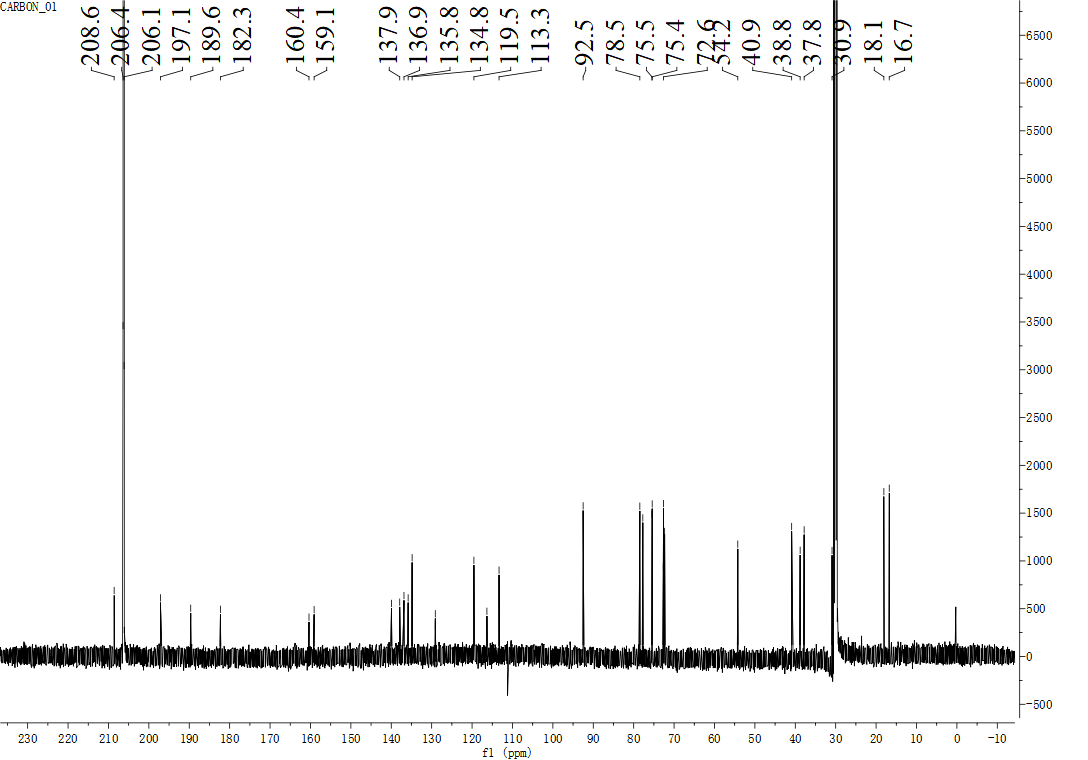


**Figure S11**. ^13^C NMR spectrum of grincamycin W (**2**) at 150 MHz in actone-*d_6_*.


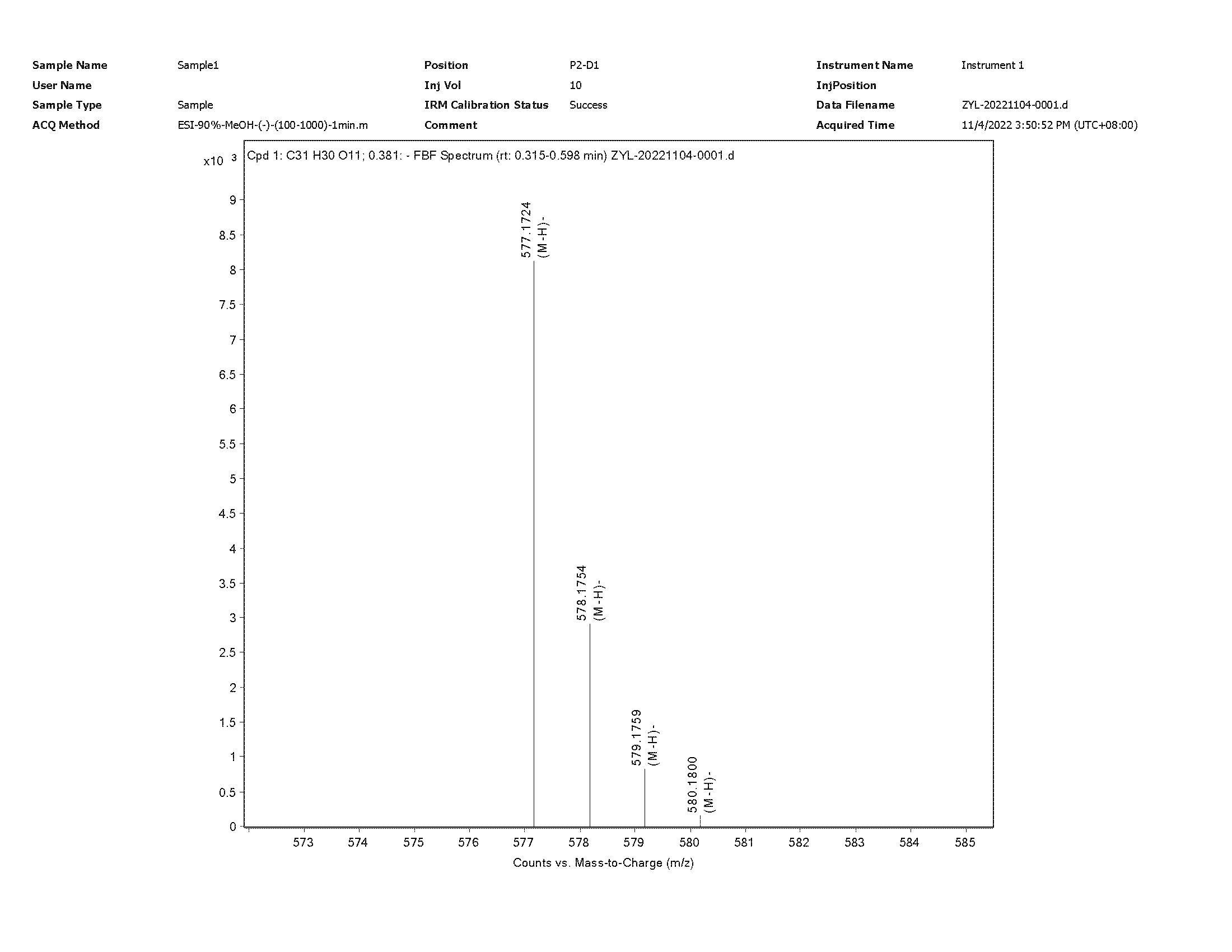


**Figure S12**. HR-ESI-MS spectrum of grincamycin W (**2**).


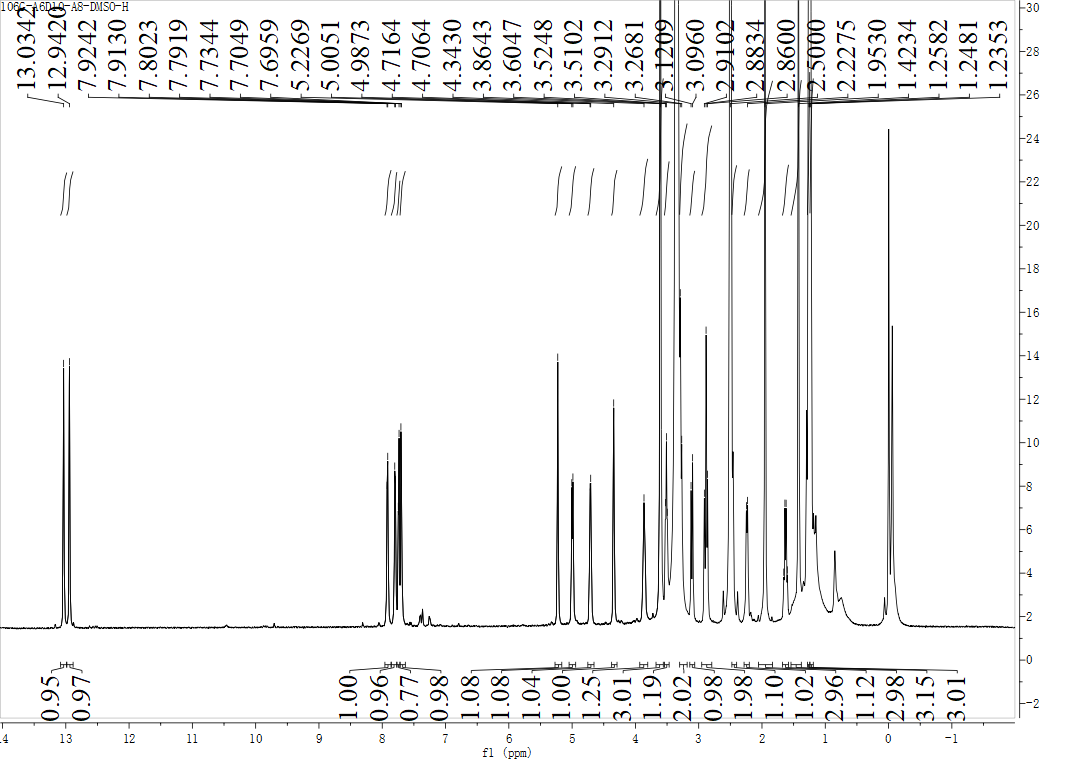


**Figure S13**. ^1^H NMR spectrum of **4A** at 600 MHz in DMSO-*d_6_*.
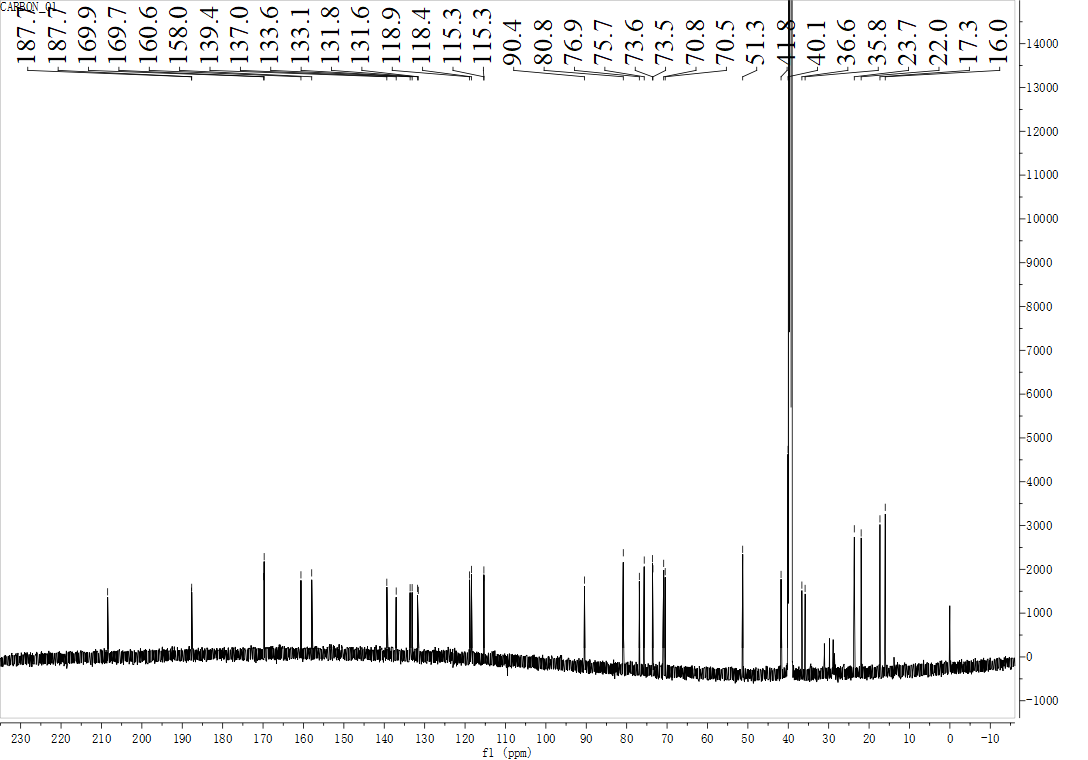


**Figure S14**. ^13^C NMR spectrum of **4A** at 150 MHz in DMSO-*d_6_*.


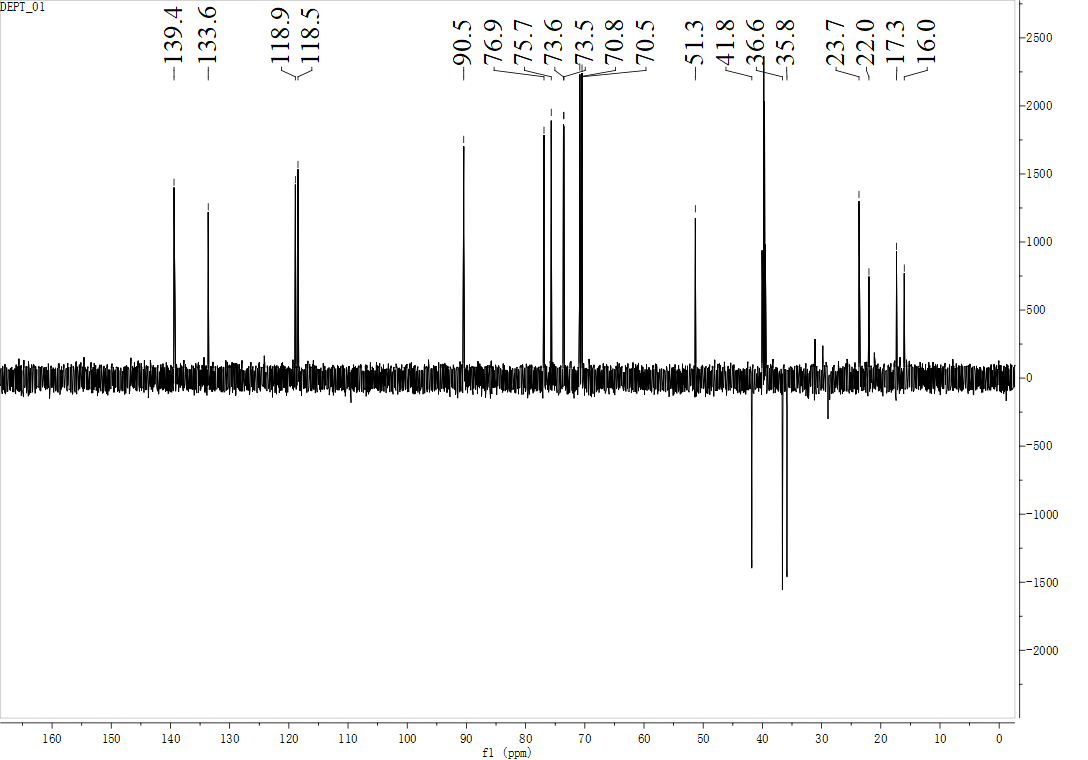


**Figure S15**. DEPT135 spectrum of **4A** at 150 MHz in DMSO-*d_6_*.


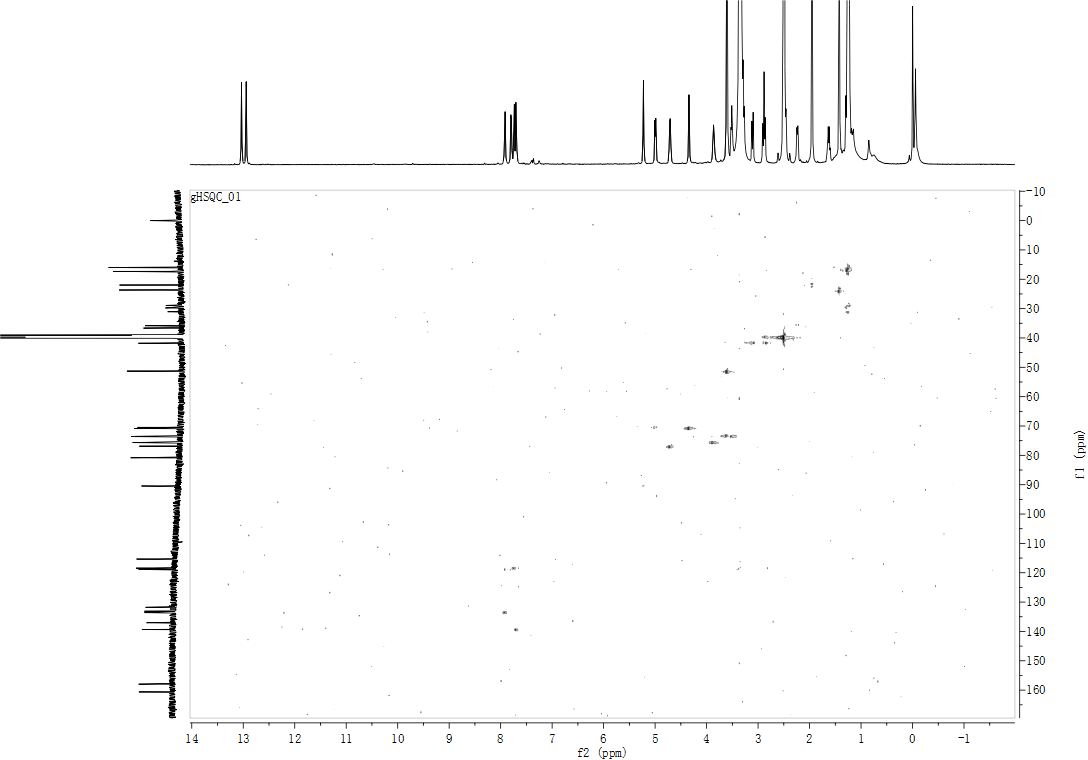


**Figure S16**. HSQC spectrum of **4A** in DMSO-*d_6_*_._


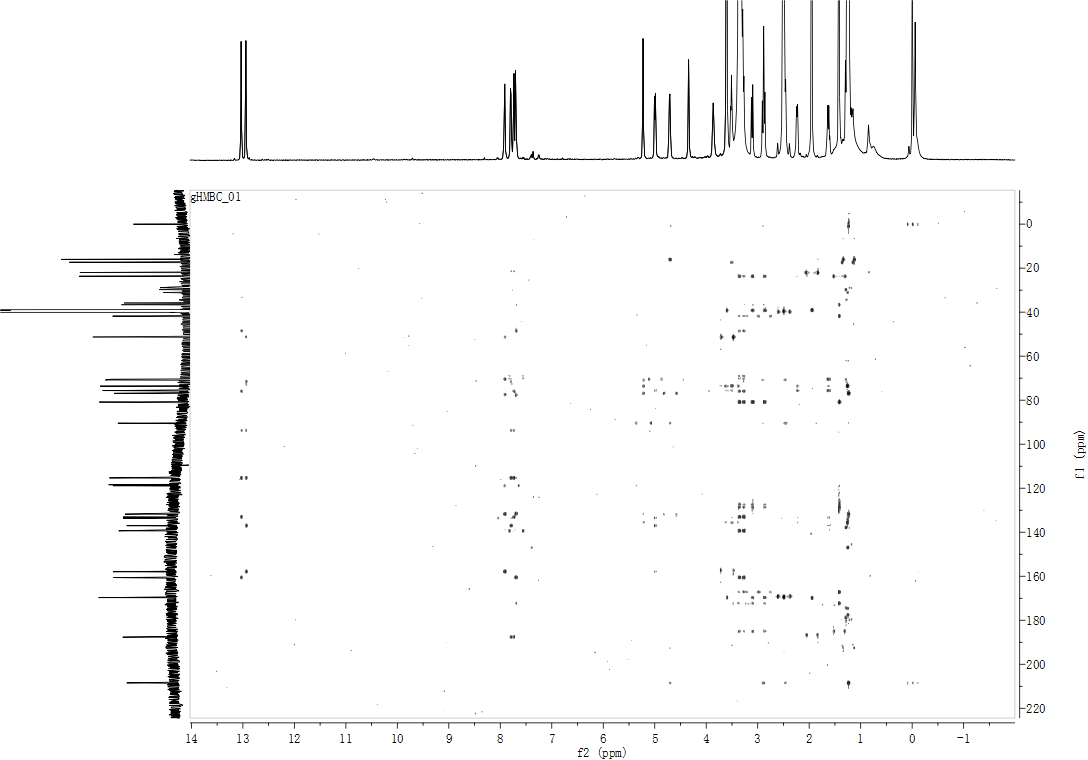


**Figure S17**. HMBC spectrum of **4A** in DMSO-*d_6_*_._


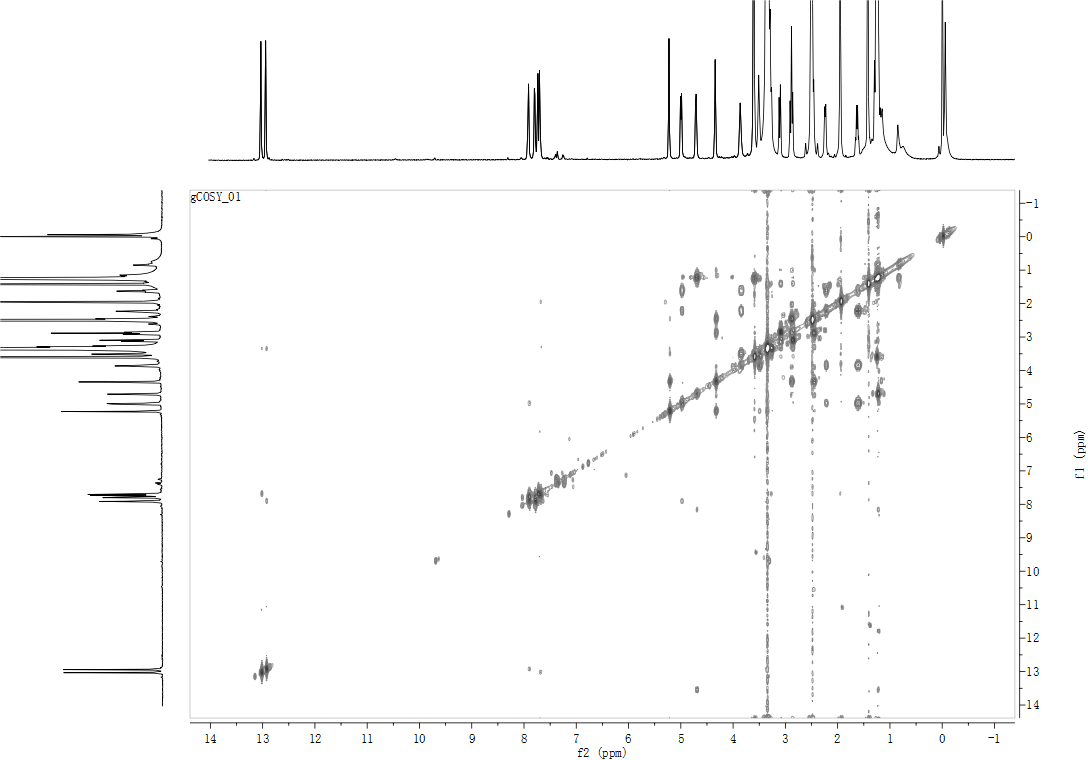


**Figure S18** ^1^H-^1^H COSY spectrum of **4A** in DMSO-*d_6_*_._


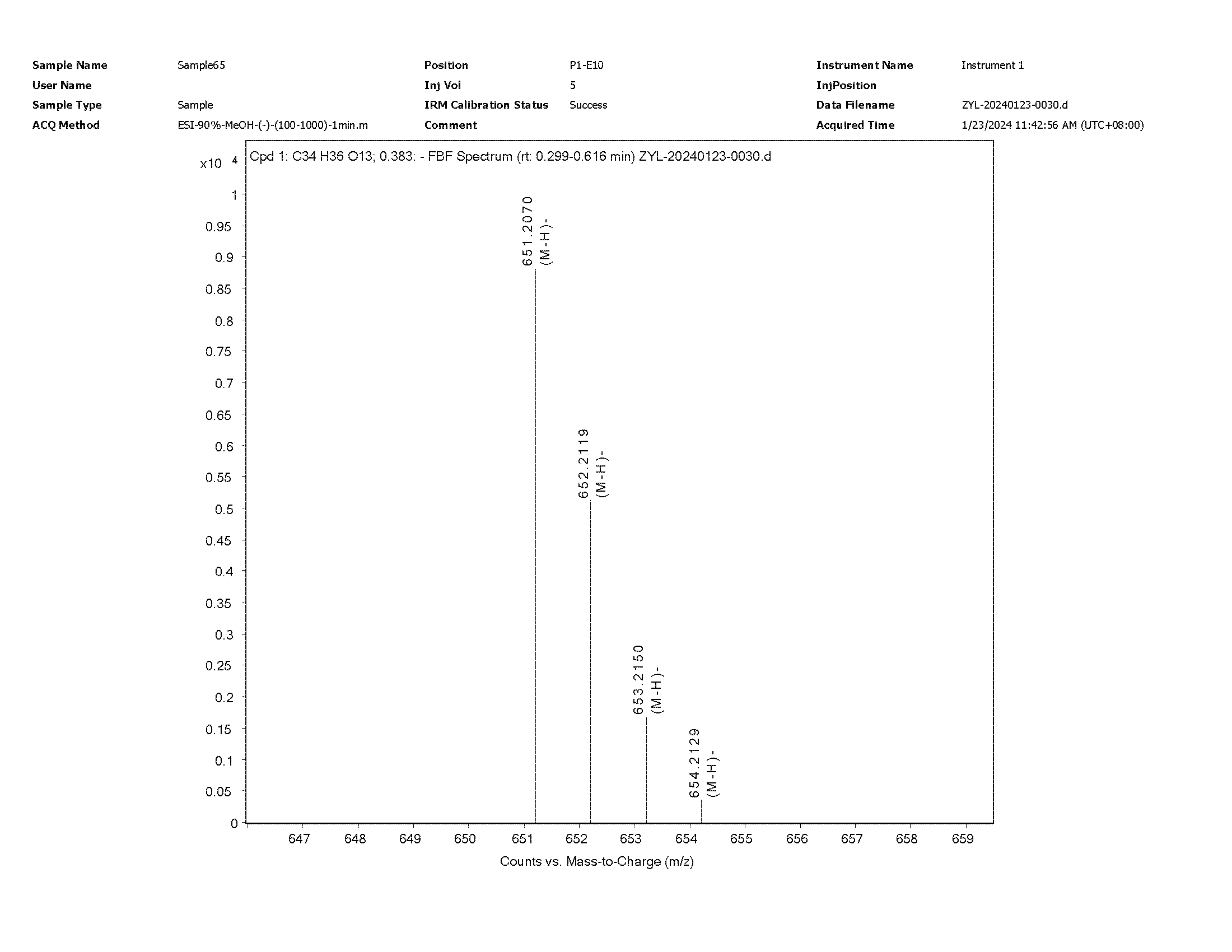


**Figure S19**. HR-ESI-MS spectrum of **4A**.


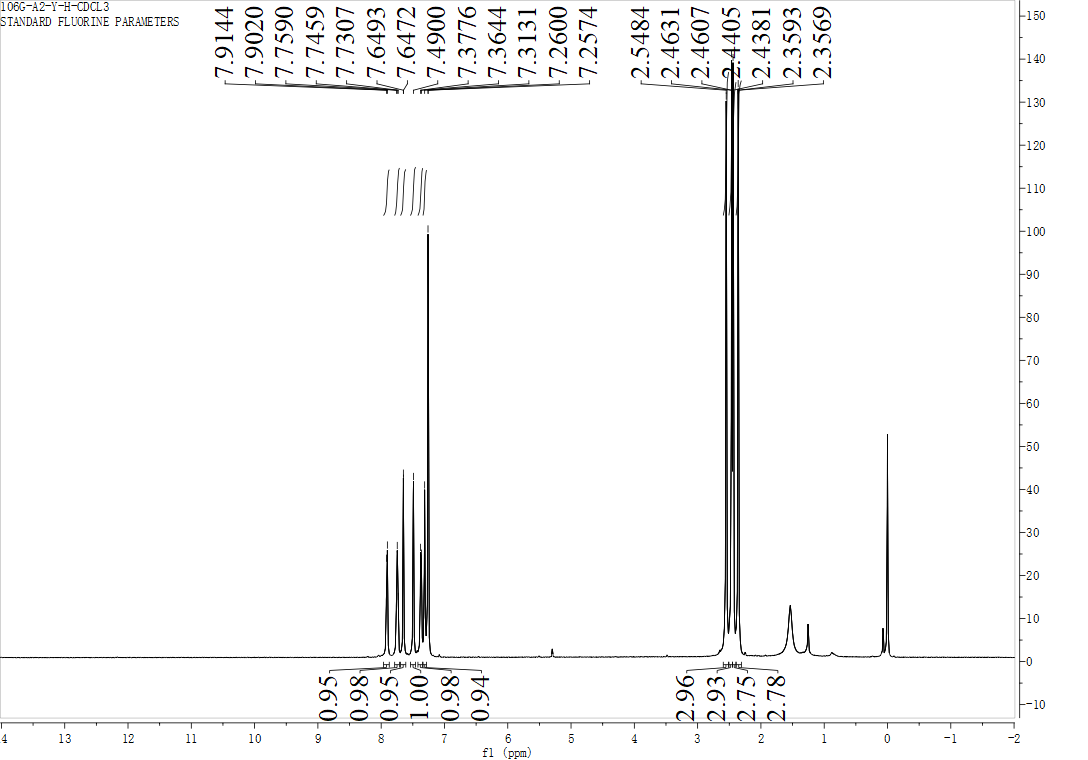


**Figure S20**. ^1^H NMR spectrum of **5A** at 600 MHz in CDCl_3_.


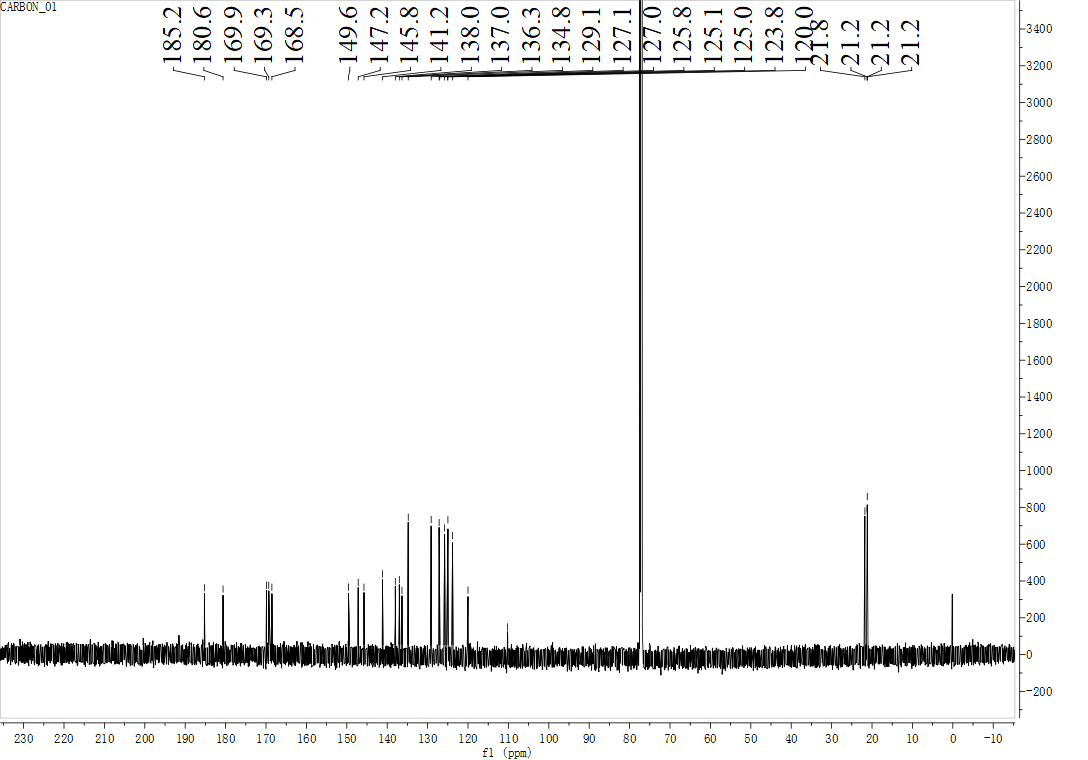


**Figure S21**. ^13^C NMR spectrum of **5A** at 150 MHz in CDCl_3_.


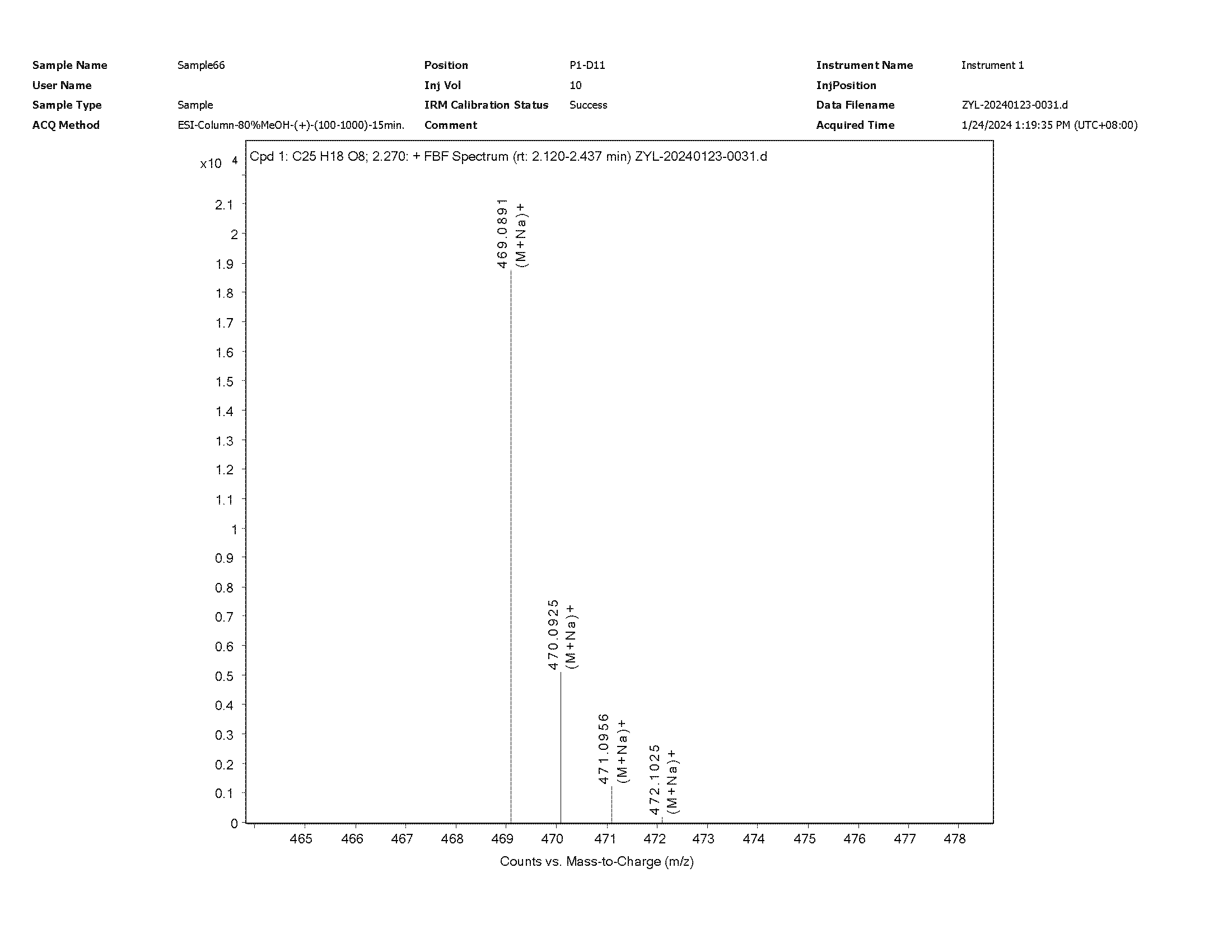


**Figure S22**. HR-ESI-MS spectrum of **5A**.


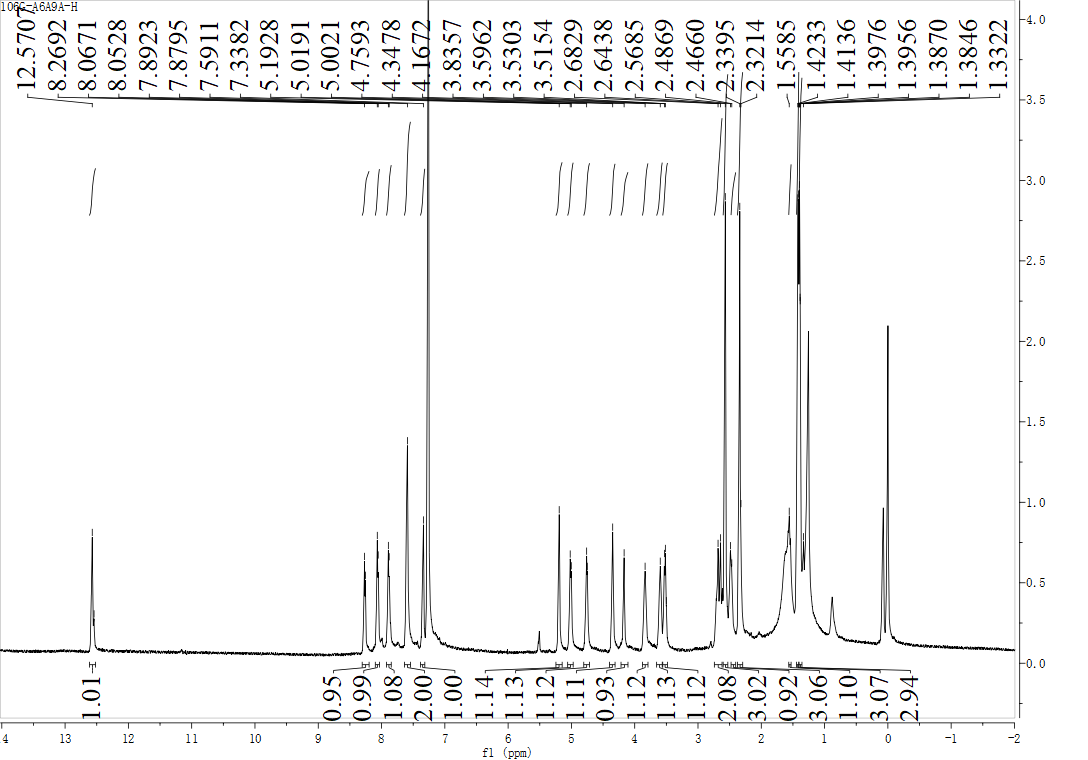


**Figure S23**. ^1^H NMR spectrum of **6A** at 600 MHz in CDCl_3_.


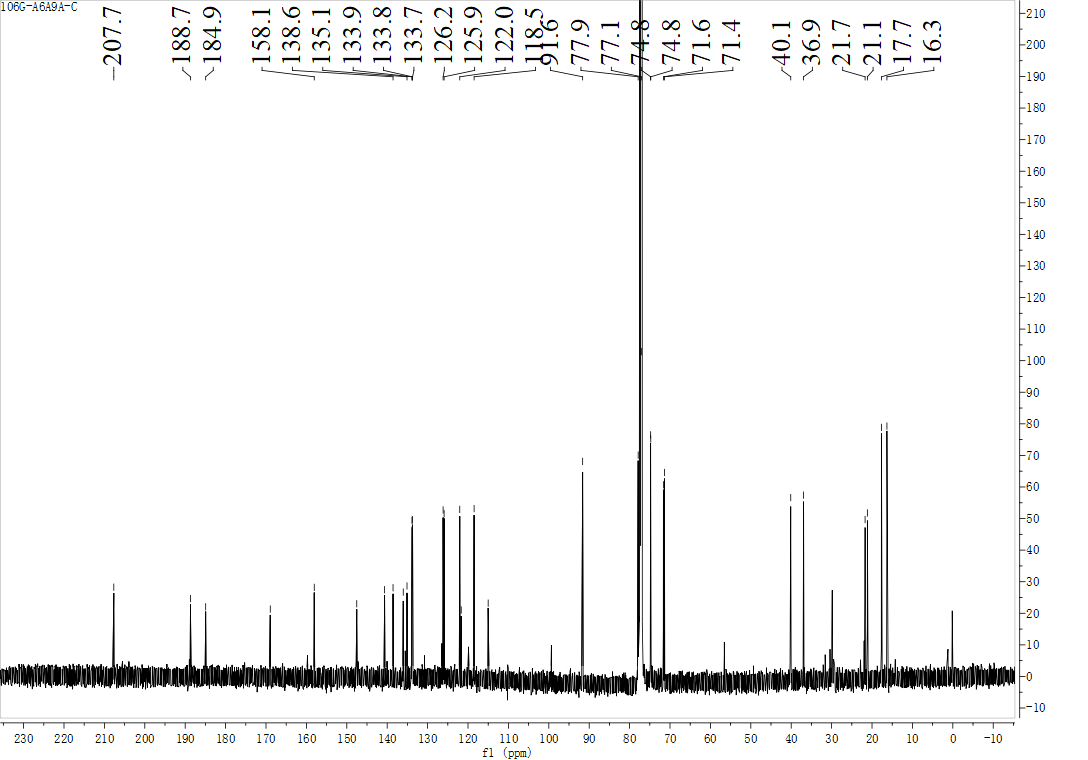


**Figure S24**. ^13^C NMR spectrum of **6A** at 150 MHz in CDCl_3_.


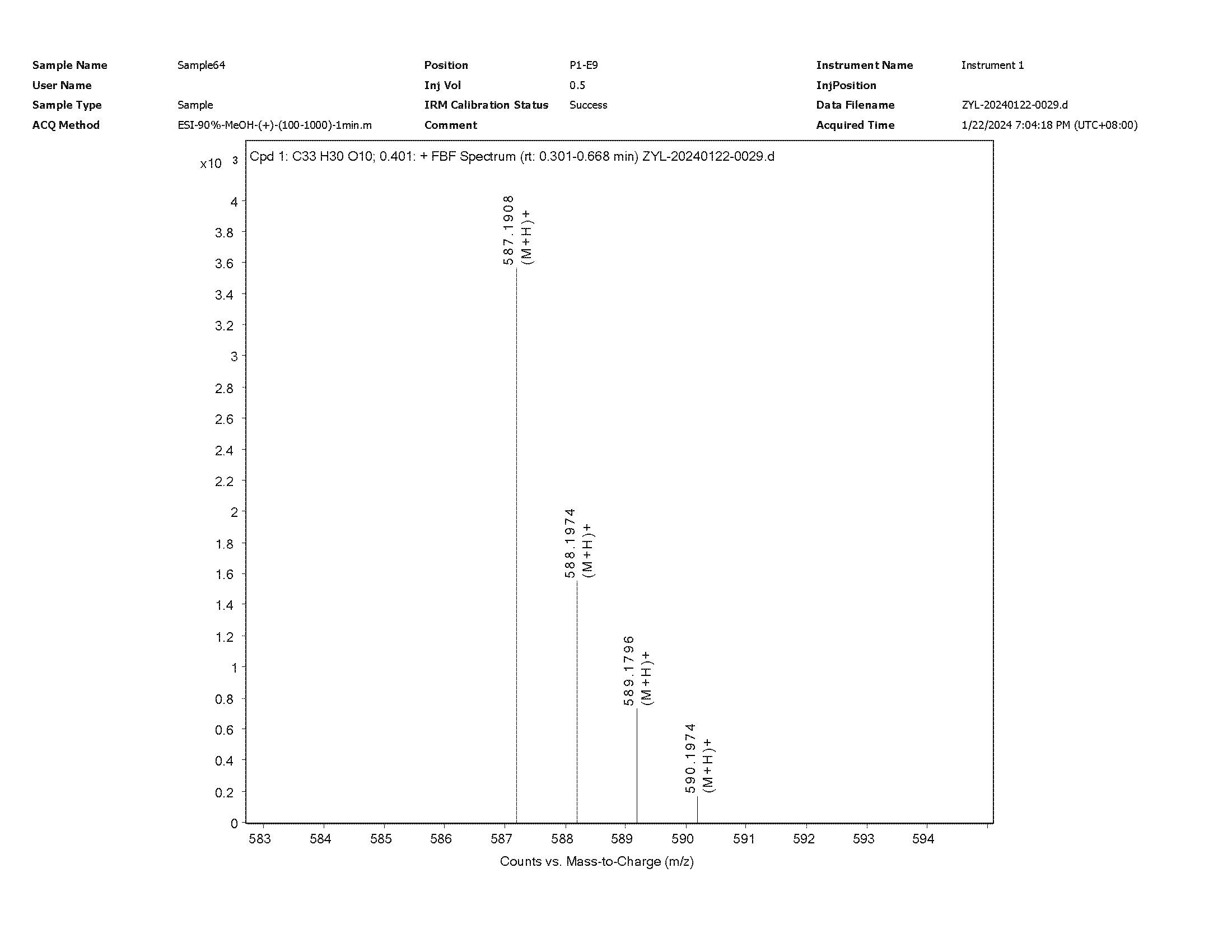


**Figure S25**. HR-ESI-MS spectrum of **6A**.


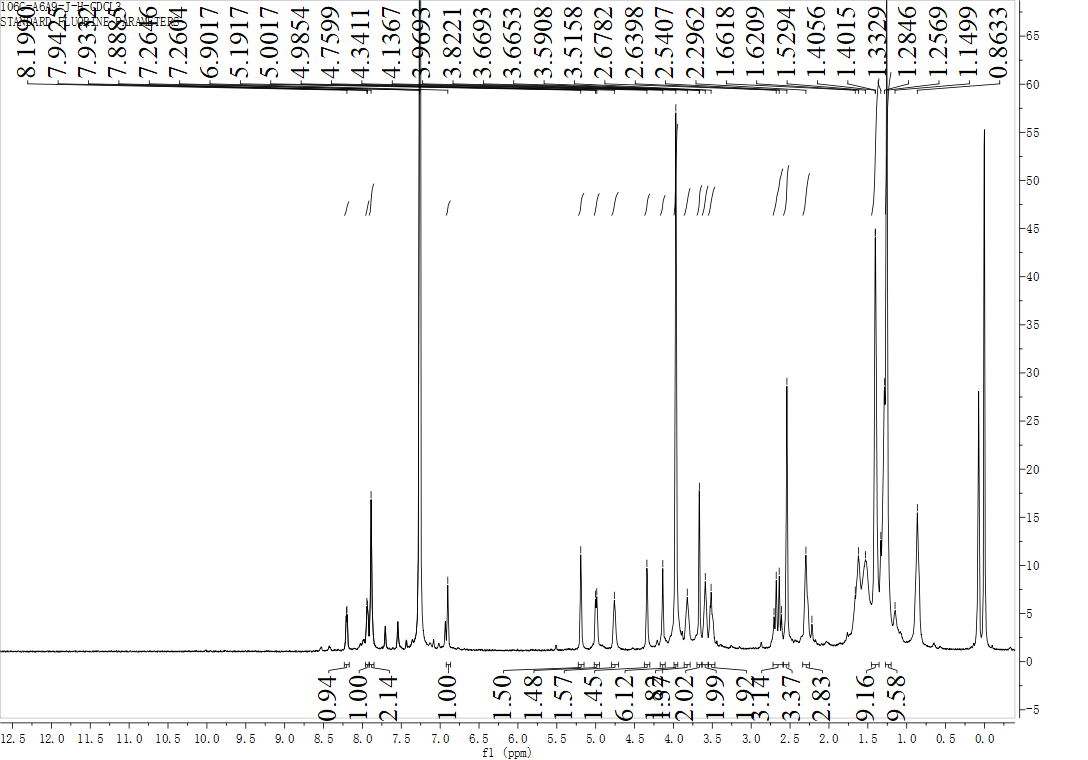


**Figure S26**. ^1^H NMR spectrum of **6B** at 600 MHz in CDCl_3_.


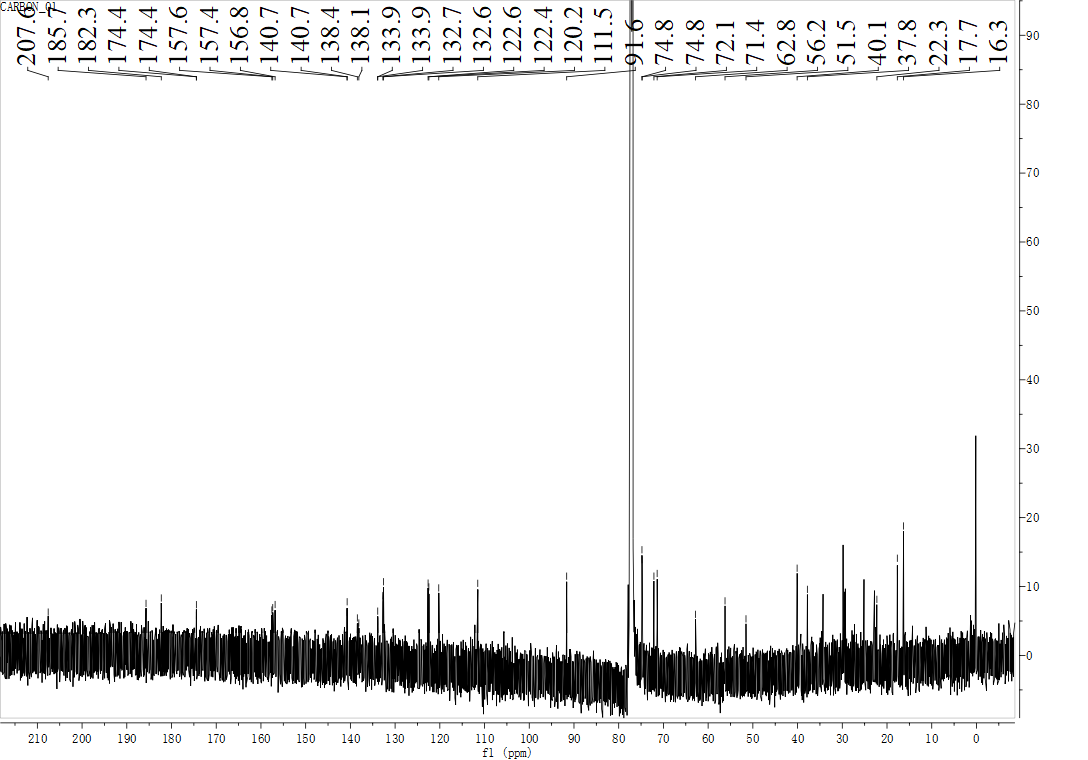


**Figure S27**. ^13^C NMR spectrum of **6B** at 150 MHz in CDCl_3_.


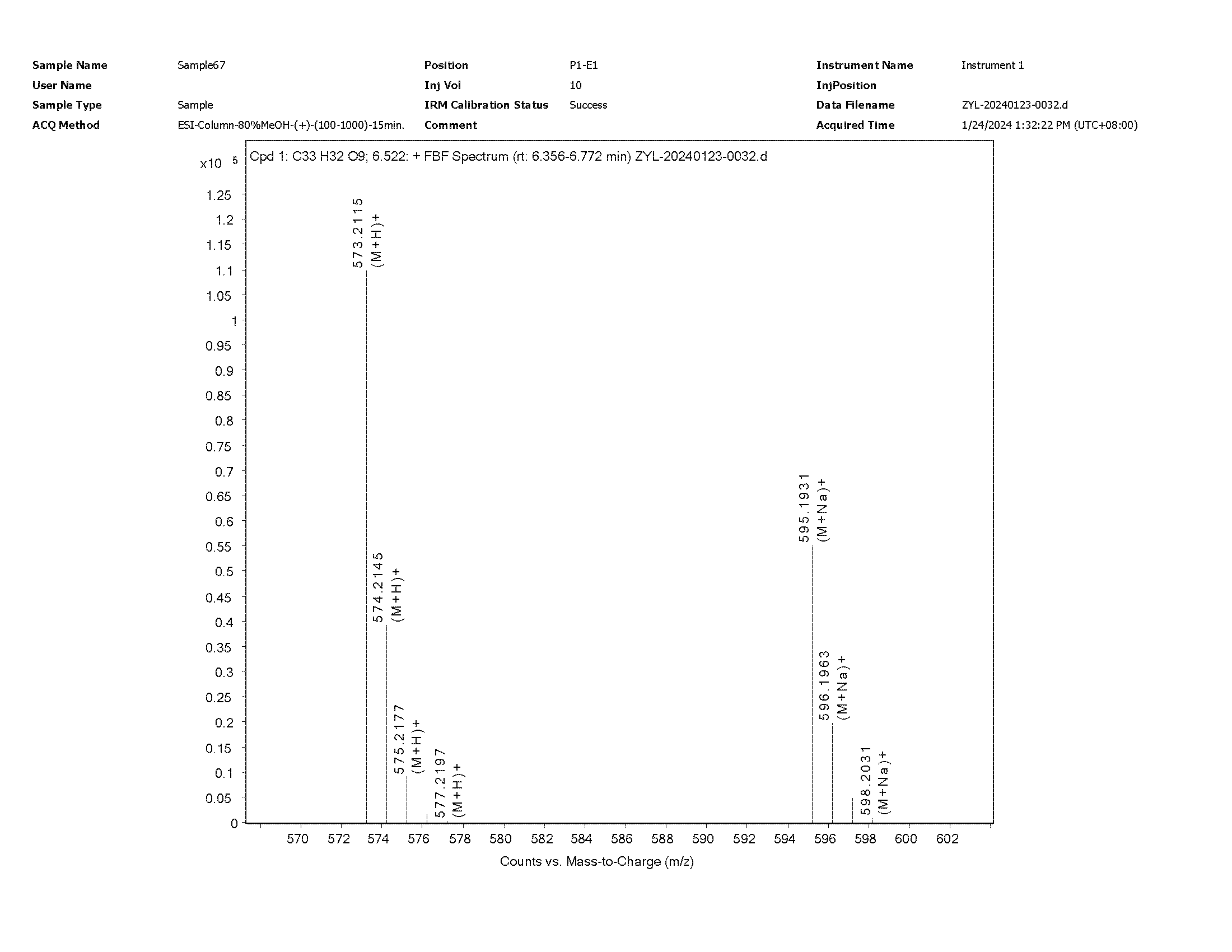


**Figure S28**. HR-ESI-MS spectrum of **6B**.


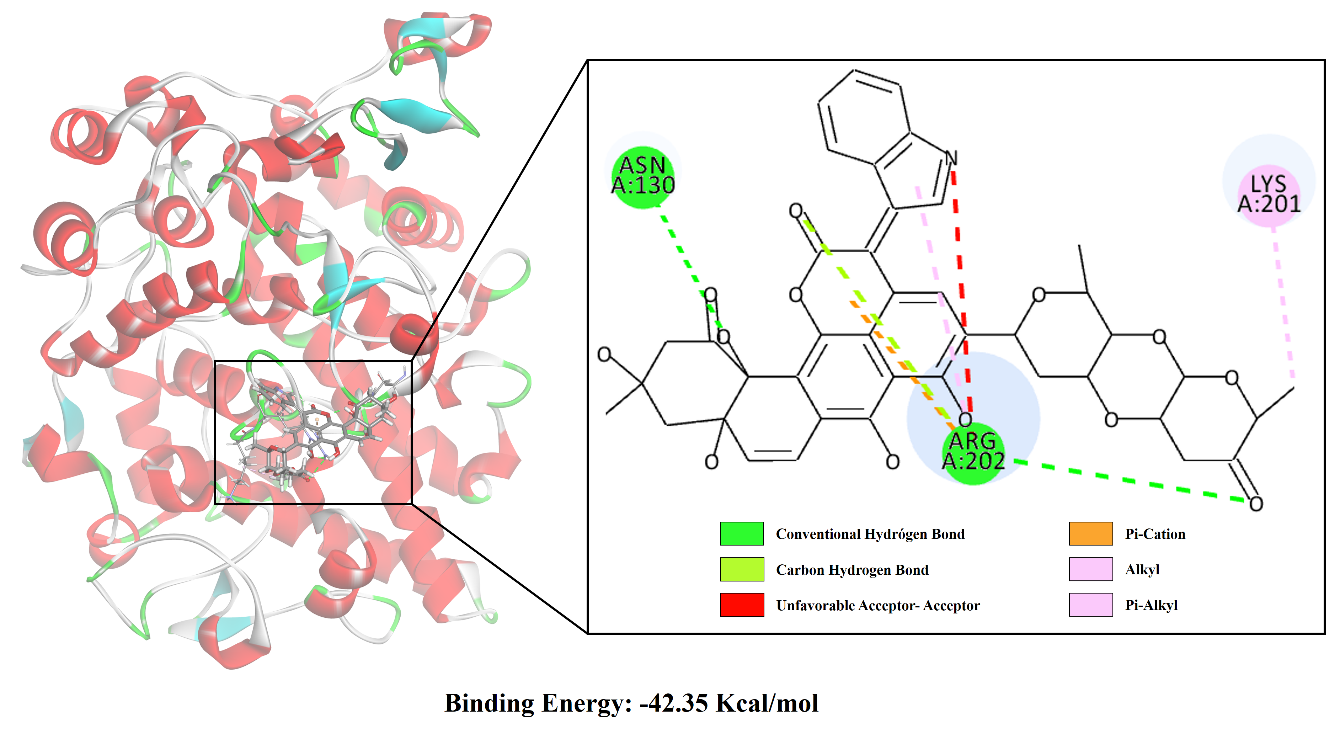


**Figure S29**. Molecular docking model of **1** into COX-2 binding site.


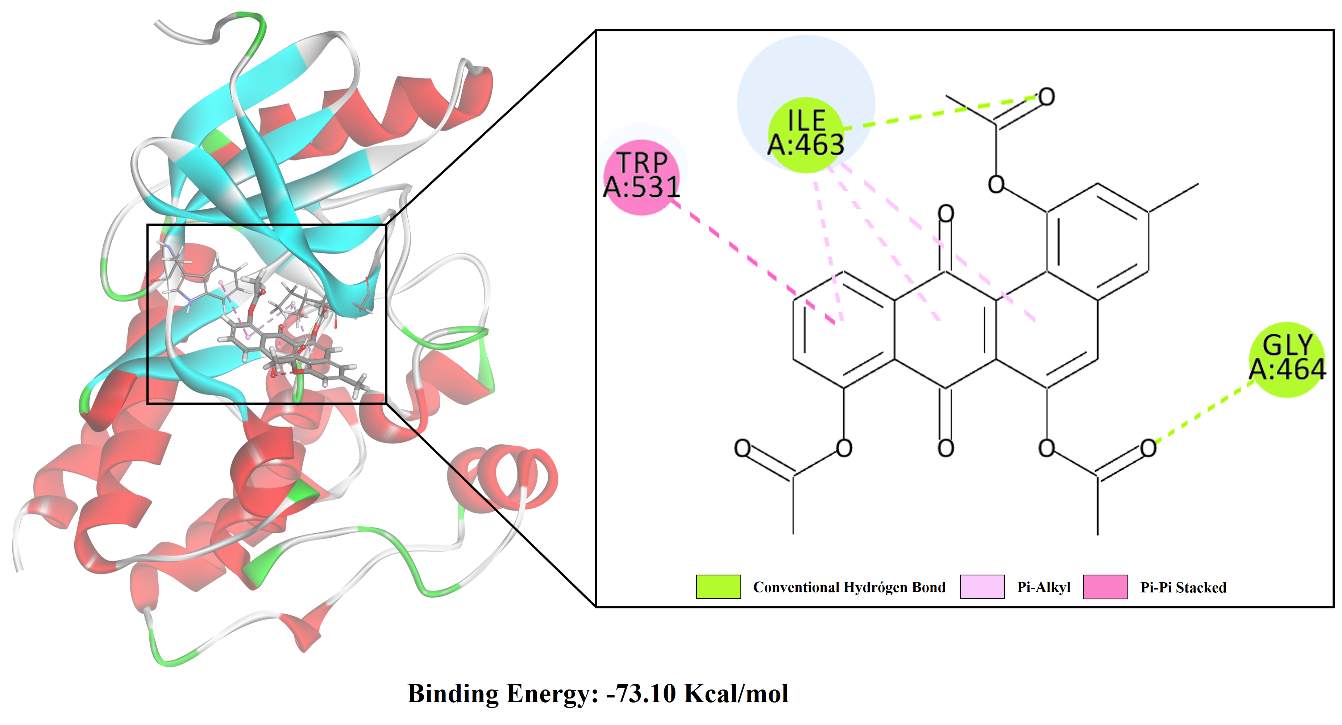


**Figure S30**. Molecular docking model of **5A** into BRAF^V600E^ binding site.
